# Surface Charge Can Modulate Phase Separation of Multidomain Proteins

**DOI:** 10.1021/jacs.3c12789

**Published:** 2024-01-23

**Authors:** Jonggul Kim, Sanbo Qin, Huan-Xiang Zhou, Michael K. Rosen

**Affiliations:** † Department of Biophysics, University of Texas Southwestern Medical Center, Dallas, Texas 75390, United States; ‡ Howard Hughes Medical Institute, Dallas, Texas 75390, United States; § Department of Chemistry, University of Illinois Chicago, Chicago, Illinois 60607, United States; ∥ Department of Physics, University of Illinois Chicago, Chicago, Illinois 60607, United States

## Abstract

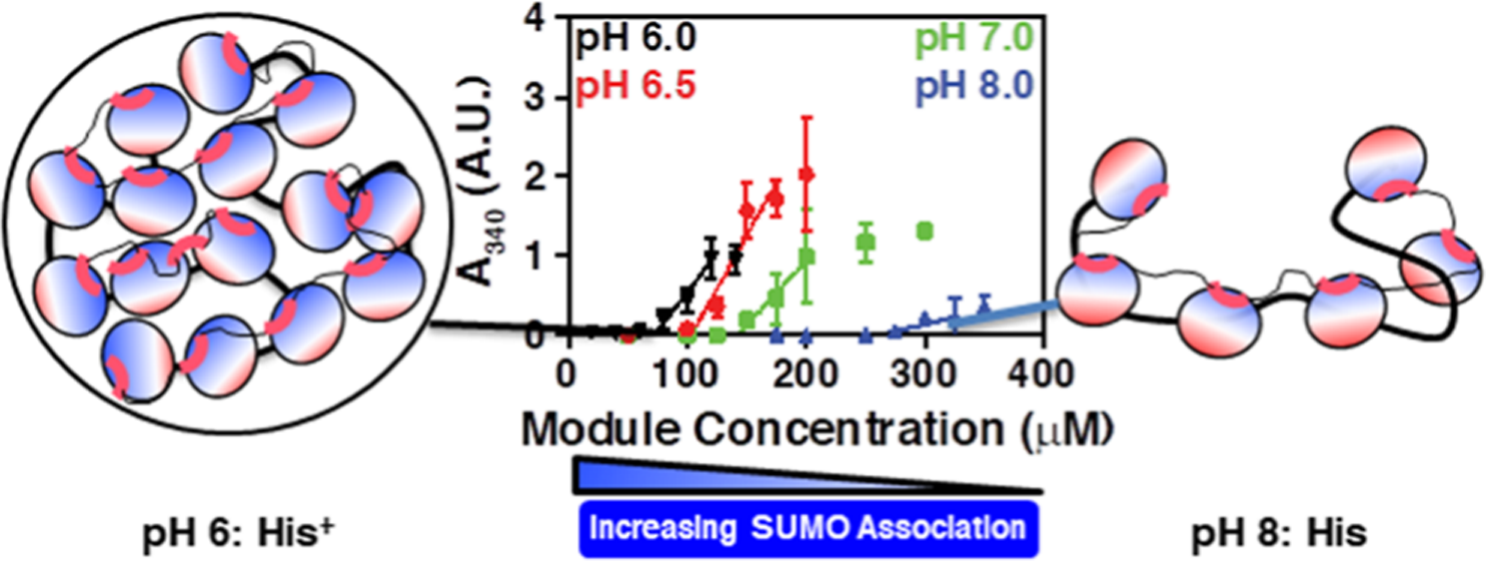

Phase separation has emerged as an important mechanism explaining the formation of certain biomolecular condensates. Biological phase separation is often driven by the multivalent interactions of modular protein domains. Beyond valency, the physical features of folded domains that promote phase separation are poorly understood. We used a model system—the small ubiquitin modifier (SUMO) and its peptide ligand, the SUMO interaction motif (SIM)—to examine how domain surface charge influences multivalency-driven phase separation. Phase separation of polySUMO and polySIM was altered by pH via a change in the protonation state of SUMO surface histidines. These effects were recapitulated by histidine mutations, which modulated SUMO solubility and polySUMO–polySIM phase separation in parallel and were quantitatively explained by atomistic modeling of weak interactions among proteins in the system. Thus, surface charge can tune the phase separation of multivalent proteins, suggesting a means of controlling phase separation biologically, evolutionarily, and therapeutically.

## Introduction

The organization of eukaryotic cells is dependent on the compartmentalization of biomolecules through organelles, which perform unique functions. In recent years, biomolecular condensates have emerged as widespread, functionally important compartments in diverse cell types and organisms. In contrast to canonical organelles, condensates concentrate molecules, including proteins, RNA, and small molecules, without a surrounding membrane.^1,2^ Many condensates dynamically exchange material with the environment and exhibit physical properties of viscous liquids. Condensates are typically enriched in multivalent macromolecules—those containing multiple weakly adhesive elements connected by flexible linkers—and a variety of studies have shown that the assembly of such molecules is essential to forming condensates. Often this assembly leads to phase separation, and many condensates have been proposed to form through this mechanism, both in biochemical reconstitutions and in cells.^3−7^

Many phase-separating proteins contain multiple folded domains that are connected and flanked by intrinsically disordered regions (IDRs).^8^ Molecular dissections have demonstrated that both the folded domains and IDRs often contribute to the drive for phase separation.^9−11^ IDRs have been extensively studied, and their features promoting phase separation include cation–π interactions, π–π interactions, charge patterning, and backbone and side chain hydrogen bonding.^12−17^ The typically higher affinity interactions of modular domains with either other domains or short linear interaction motifs (SLIMs) often are viewed as contributing primarily to oligomerization, which together with IDR interactions decreases solubility, promoting phase separation.^1^ Beyond valency and ligand affinity, however, the properties of the modular domains that promote phase separation in these systems have not been extensively studied.

It has long been observed that single-domain proteins can undergo phase separation at very high concentrations (>5 mM, e.g., lysozyme^18^ and γ-crystallin^19,20^) driven by weak self-interactions.^21−24^ Such interactions contrast with both the stronger binding of modular domains with their specific ligands and the association of IDRs in poor solvents, which allow phase separation to occur at physiological concentrations (nM–μM). Here, we ask whether weak self-interactions of modular domains, when presented in a multivalent fashion, can alter the drive for phase separation to a significant degree. If so, can this property be exploited to make rational mutations of solvent-exposed residues to alter phase separation in a manner independent of IDR solubility and multivalent interactions? To address these questions, we used a synthetic protein system based on multivalent interactions between the small ubiquitin-like modifier (polySUMO) and the SUMO interaction motif (polySIM). We found that the solubility of the SUMO1 domain (SUMO hereafter), as assessed through the second scattering virial coefficient (*A*_2_), increases with increasing pH and that this property correlates with a weakened drive for polySUMO–polySIM phase separation. Site-directed mutagenesis suggests that the pH-dependent change in SUMO solubility likely derives from the change in the protonation state of surface histidines. Correspondingly, histidine mutations, when presented in a multivalent fashion in polySUMO, can significantly alter the phase separation. We gained insight into the molecular basis for the effects of pH and surface mutations on SUMO solubility and polySUMO–polySIM phase separation through atomistic modeling of weak interactions between SUMO domains and between SUMO and SIM, respectively. The modeling suggested intermolecular contacts that exert dominant influences on solubility and phase separation, with the effects of the proposed mutations validated by experimental measurements. Our results indicate that the weak attraction, as opposed to high-affinity stereospecific binding, between folded domains and SLIMs, along with weak self-association of folded domains, can strongly affect the formation of biomolecular condensates, suggesting avenues to manipulate condensates in a manner independent of linker solubility and specific biomolecular interactions.

## Results

### PolySUMO–PolySIM Phase Separation Is pH Dependent

**Figure 1:**
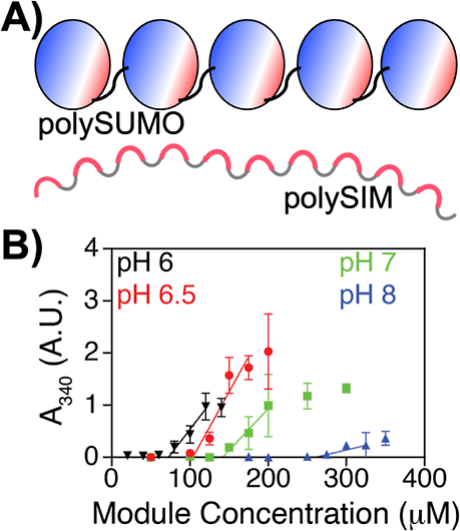
pH-dependent phase separation of polySUMO–polySIM. (A) Cartoon representation of polySUMO and polySIM. (B) Turbidity (*A*_340_) of polySUMO–polySIM solutions at pH 6.0–8.0. The phase-separation threshold was determined by extrapolating the three points above the first concentration above an *A*_340_ value of 0.1 to the *x*-intercept. Each data point represents the mean ± SD of three independent measurements.

Both SUMO and SIM are found in proteins that localize to biomolecular condensates, including PML nuclear bodies and stress granules.^25^ SUMO isoforms are often attached to their targets in linear chains, and interactions of such chains with multi-SIM sequences have been proposed to promote biologically relevant phase separation.^26^ We previously demonstrated that a synthetic protein consisting of five SUMO domains (polySUMO; Figure 1A) can phase-separate in biochemical systems and in cells when mixed with a protein consisting of 10 SIMs (polySIM; Figure 1A) derived from the PIASx protein.^27^ In the course of this work, we found that at pH 7, the polySUMO phase separates at a module concentration of ∼150 μM when mixed with an equimolar module concentration of polySIM, as assessed by turbidity measurements at 340 nm (Figure 1B). However, at pH 8, a module concentration of nearly 300 μM is needed to observe phase separation. Likewise, when the experiments were repeated at pH 6.5 and 6, the phase separation threshold decreased to module concentrations of ∼100 and ∼75 μM, respectively. Thus, phase separation driven by multivalent SUMO–SIM interactions is pH dependent, showing an ∼4-fold range of concentration threshold between pH 6 and 8.

### Weak SUMO Self-Association, but Not High-Affinity SUMO–SIM Binding, Is pH Dependent

Previous studies have suggested that solvation effects on intrinsically disordered linkers can play a strong role in promoting phase separation of multivalent systems.^9,11,28^ However, the intermodule linkers in polySUMO and polySIM consist of a tandem (Gly–Gly–Ser)_4_ repeat sequence. Given this amino acid composition, it is unlikely that linker solvation would change substantially between pH 6 and 8. We thus considered whether changes to the SUMO–SIM interaction or to SUMO itself might account for the pH dependence of polySUMO–polySIM phase separation.

**Figure 2:**
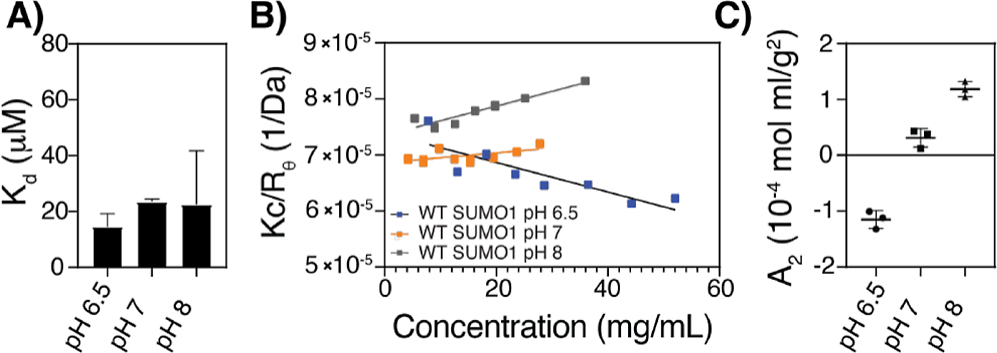
Second virial coefficient, not binding affinity, increases in a pH-dependent manner. (A) *K*_D_ of SUMO for SIM measured by isothermal titration calorimetry at 25 °C. Bars show mean ± SE of two or three replicate measurements. (B) Static light scattering of WT SUMO at 25 °C as a function of pH and mass concentration. (C) *A*_2_ values determined from the slope of scattering intensity versus mass concentration in panel (B). Bars show mean ± SE of three replicate measurements.

In multidomain systems, the affinity between the modular elements affects the degree of assembly (which in turn affects phase separation).^29−31^ To determine whether this factor could account for the pH dependence here, we measured the affinity of SUMO for SIM using isothermal titration calorimetry (ITC; Figure S1). The *K*_D_ values determined at pH 6.5, 7, and 8 were very similar, ranging between 15 and 20 μM, and are statistically indistinguishable (Figure 2A). Thus, some feature of the system other than the stereospecific binding of SUMO to SIM causes pH dependence of phase separation.

At high concentrations (>100 mg/mL) and in low salt conditions, many single-domain proteins can undergo phase separation driven by weak, nonspecific self-interactions.^18,19,32,33^ Thus, we next considered whether SUMO might weakly self-associate and, if so, whether these interactions are pH dependent. To quantify self-interactions, we used static light scattering (SLS) to determine the second scattering virial coefficient (*A*_2_) of a single SUMO domain (Figure 2B). The *A*_2_ value describes the first-order deviation from ideal solution behavior, which arises from self-interaction at the binary level and is determined by the orientationally averaged potential of mean force between a pair of protein molecules of the same species.^34^ A negative *A*_2_ value indicates that the protein has energetically favorable self-association, while a positive value indicates repulsion. *A*_2_ is quantitatively related to the solubility of the protein^35,36^ and has been used previously to predict the liquid–liquid phase separation and crystallization behaviors of folded proteins.^24,37−39^ Because of these relationships, we use *A*_2_ here as an indicator of SUMO solubility. We found that the *A*_2_ value of SUMO is negative at pH 6.5 (*A*_2_ = −1.2 × 10^–4^ mol mL g^–2^), indicating self-association and lower solubility. *A*_2_ progressively increases to a positive value at pH 8 (*A*_2_ = 1.2 × 10^–4^ mol mL g^–2^), indicating self-repulsion and higher solubility at the higher pH (Figure 2C). Although we were unable to measure *A*_2_ at pH 6 due to precipitation at concentrations needed for the measurement, in this acidic condition, SUMO formed large soluble aggregates that were absent at higher pH values, consistent with strong self-association (Figure S2). Together, the data show that pH-dependent changes in SUMO self-association and solubility, but not SUMO–SIM stereospecific binding or linker solvation, parallel the pH dependence of polySUMO–polySIM phase separation. Moreover, a similar pH dependence of self-association (along with a lack of pH dependence in SIM affinity) was observed for the SUMO3 isoform (Figure S3A,B), suggesting that self-association is a conserved feature of SUMO proteins that might be functionally relevant in biological systems.^40^ A strong dependence of *A*_2_ on salt concentration points to the importance of electrostatic attraction in SUMO self-association (Figure S3C).

### Surface Histidines Account for the pH Dependence of SUMO Self-Association

**Figure 3:**
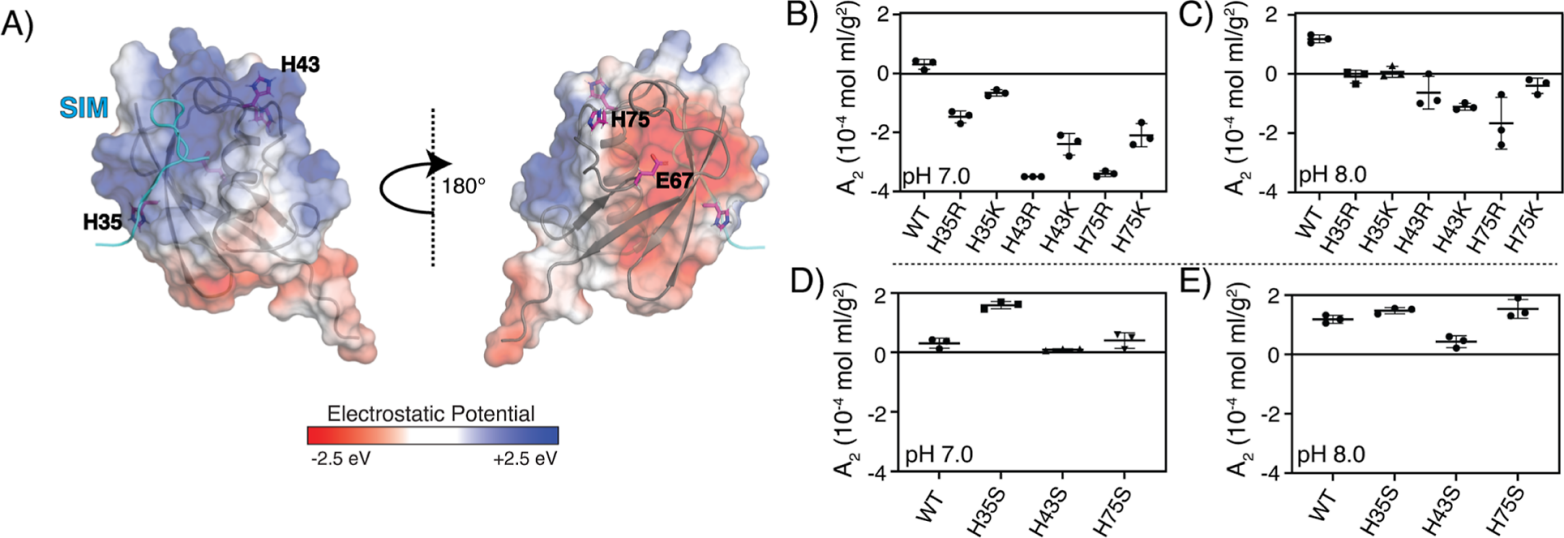
pH-dependent *A*_2_ can be mimicked by site-directed mutagenesis. (A) Surface electrostatic potential of SUMO (PDB: 2ASQ) in two orientations at pH 7. (B,C) *A*_2_ values of WT and histidine-to-arginine/lysine mutants of SUMO were determined at pH 7 (B) and 8 (C). (D,E) *A*_2_ values of WT and histidine-to-serine mutants of SUMO were determined at pH 7 (D) and 8 (E).

Given the pH range in which *A*_2_ is altered, we hypothesized that the change in self-association of SUMO was due to a change in the protonation state of histidine side chains. SUMO contains three histidine residues, H35, H43, and H75, all of which are surface-exposed (Figure 3A). H43 and H75 are proximal to each other (7.7 Å Cβ–Cβ distance in Protein Data Bank (PDB) entry 2ASQ(41)) in adjacent loops at one end of the protein; H35 is toward the opposite end. H35 and H43 flank a large basic patch that contains the SIM binding site; hence, electrostatic attraction contributes to the binding affinity of the highly acidic SIM peptide.^41^ On the opposite face of SUMO is a large acidic patch, with E67 near its center. H75 is between the two oppositely charged patches. To learn which of these histidine residues might contribute to the pH dependence of *A*_2_, we first mutated each individually to either arginine or lysine, to mimic the protonated (low pH) state of the wild-type (WT) protein, and measured *A*_2_ at pH 7 (Figure S4) and 8. At pH 7, all Lys and Arg mutants showed negative *A*_2_ values, indicating greater self-association than WT SUMO, which has a slightly positive *A*_2_ (Figure 3B). This result indicates that protonating the histidines promotes self-association since at pH 7, the histidine side chains are only partially protonated (Figure S5), while Lys and Arg are fully protonated. Larger effects on *A*_2_ can be seen for the H43 and H75 mutants than for the H35 mutant, suggesting that the former two sites may play a greater role in pH-dependent self-association of the WT protein. At all sites, the Arg mutants had lower *A*_2_ values than their Lys counterparts, consistent with studies indicating that arginine is often a stronger driver of weak interactions than lysine.^12,14^ At pH 8, the Arg and Lys mutants all retained their less positive *A*_2_ values than WT SUMO, again with sites 43 and 75 showing greater effects than 35 (Figure 3C). Thus, protonating even a single histidine can increase the self-association of SUMO.

We next examined the effects of mutating histidines to serines, mimicking a constitutively deprotonated state at each position. At pH 7, the mutants had *A*_2_ values relatively close to that of the WT protein, except for H35S (Figure 3D), whose *A*_2_ was more positive (1.6 × 10^–4^ vs 0.3 × 10^–4^ mol mL g^–2^ for WT) and very similar to that of WT at pH 8 (Figure 3E). This suggests that deprotonation of site 35 at the higher pH plays a dominant role in reducing self-association in SUMO. At pH 8, the *A*_2_ value of WT was similar to that of H35S and statistically identical to that of H75S (*p* > 0.05, Figure S6), consistent with full deprotonation of histidines in WT SUMO at this pH; the *A*_2_ value of H43S for some reason did not reach the WT level. Together, these observations suggest that when the pH decreases, protonation of all of the histidine residues contributes to SUMO self-association; when the pH increases, deprotonation of histidine 35 plays the most important role in reducing SUMO self-association.

To address whether protonation effects are additive between residues, we generated an H35R/H43R double mutant and measured its *A*_2_ values at pH 7 and 8 (Figure S7). The pH dependence was reduced in this mutant, as would be expected since only one of the three sites remains titratable; the *A*_2_ values were negative and very similar at both pH values (∼−2.5 × 10^–4^ mol mL g^–2^). Notably, at pH 8, where histidines would be nearly fully deprotonated, the double mutant had an *A*_2_ value much more negative than that of either single mutant, consistent with the cumulative effects of the two single mutations. However, at pH 7, the *A*_2_ value of the double mutant was intermediate between those of the two single mutants; we do not have a simple explanation for the apparent negative cooperativity between the two positive charges in the double mutant. Nevertheless, these observations further support the notion that changes in histidine protonation largely account for pH-dependent changes in *A*_2_.

### Computations Can Predict Energetics of SUMO Self-Association

We next sought to understand the energetic and structural bases of the pH dependence of *A*_2_, by applying a computational method called fast Fourier transform-based modeling of atomistic protein–protein interactions (FMAP).^42^ FMAP calculates the self-interaction energy, *U*(**R**,Ω), between two copies of a rigid protein (e.g., SUMO) at all relative separations (denoted by **R**) and relative orientations (denoted by Ω) and then averages over **R** and Ω to yield *A*_2_ as
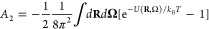
1where *k*_B_ is the Boltzmann constant and *T* is the absolute temperature. The protein is represented at the atomic level, and *U*(**R**,Ω) is the sum of contributions from all of the pairs of atoms between two copies of the protein in a configuration specified by **R** and Ω. *U*(**R**,Ω) consists of three components
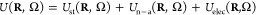
2The steric term is infinite when two atoms clash with each other (specified by a distance cutoff), and 0 otherwise. The remaining two terms operate in clash-free configurations: *U*_n-a_(**R**,Ω) models nonpolar attraction by a Lennard-Jones potential, whereas *U*_elec_(**R**,Ω) models electrostatic interactions by a Debye–Hückel potential. The effect of pH is accounted for by modeling each histidine in two protonation states, each with a set of atomic partial charges that add up to either 1 or 0.

**Figure 4:**
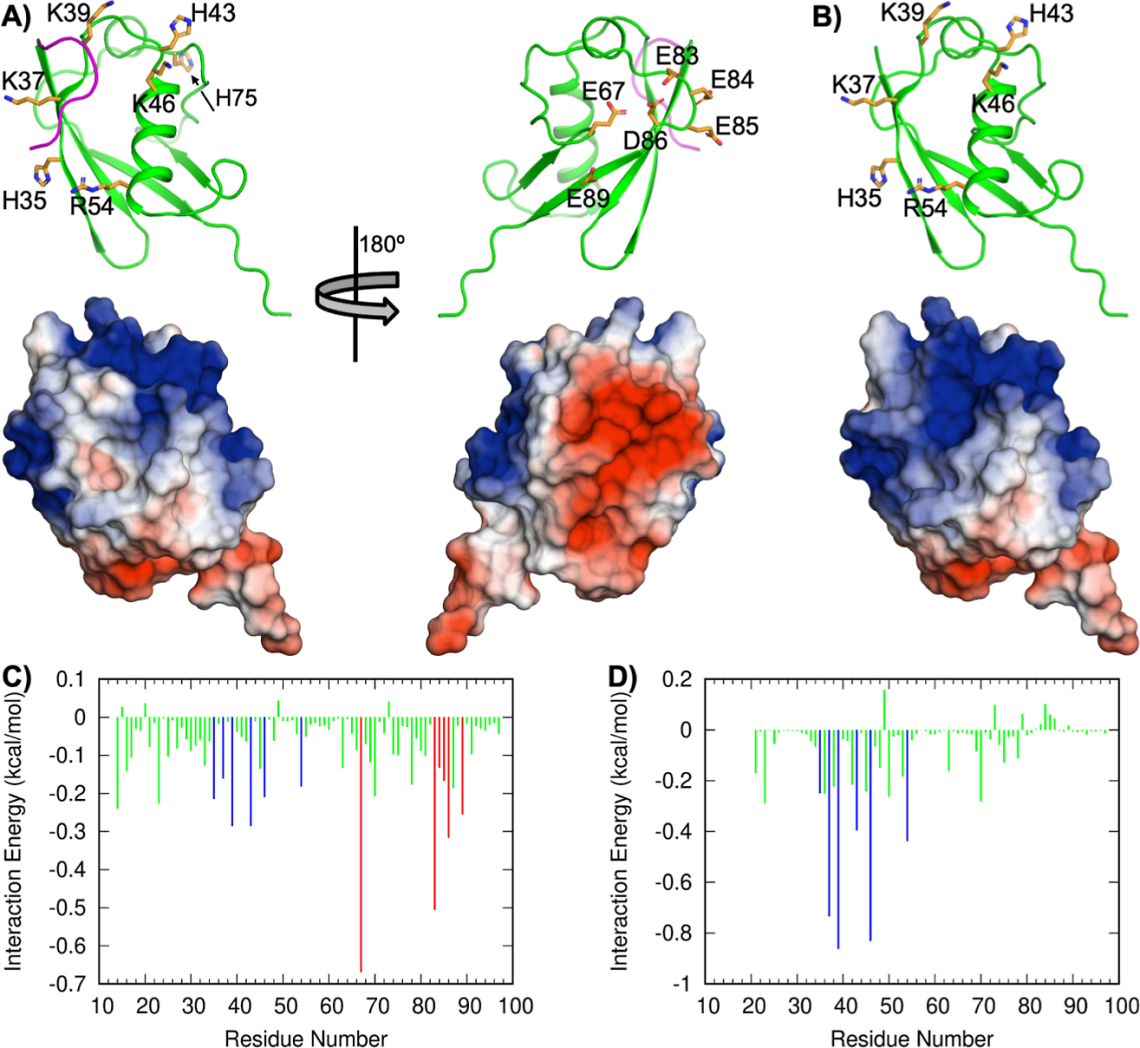
Acidic and basic residues mediate weak interactions between SUMO molecules. The results were calculated at low pH, i.e., with all of the His residues protonated. (A) Polarized charge distribution on the SUMO surface. Top: ribbon representation of SUMO residues 14–97 (backbone green, except for residues 14–20 which are magenta), showing basic and acidic side chains as sticks on opposite faces of the protein. Bottom: surface electrostatic potential showing intense positive and negative values centered around the basic and acidic clusters. (B) Similar to panel (A), except with residues 14–20 removed, mimicking the open conformation. (C) Decomposition of the binary self-interactions of SUMO determined by FMAP. Interactions between basic residues (H35, K37, K39, H43, K46, and R54; blue bars) and acidic residues (E67, E83, E84, E85, D86, and E89; red bars) make prominent contributions. (D) Decomposition of weak interactions between SUMO and SIM determined by FMAP. Basic residues (K37, K39, H43, K46, and R54; blue bars) contribute significantly to the interactions with the highly negatively charged SIM.

For FMAP calculations, we included residues G14 to G97 of SUMO, which are ordered and resolved in the crystal structure 1Y8R,^43^ assuming that the flexible N- and C-terminal residues contribute a constant amount to *A*_2_. Residues G14 to E20 (constituting the “N-tail”) adopt a structure that mimics SIM in the SUMO–SIM complex^41^ (Figure 4A, top). The N-tail is positioned at the center of the basic patch flanked by H35 and H43; the electrostatic potential around the N-tail is less positive than in the surrounding region (Figure 4A, “front” view at the bottom left). Removing the N-tail makes the electrostatic potential on the front face more positive (Figure 4B, bottom). We refer to the conformation with the N-tail bound as “closed” and the conformation with the N-tail removed as “open”. The open conformation likely predominates in the presence of SIM, which should compete with the N-tail for binding to the SUMO domain (see below).

*A*_2_ is determined by the self-interaction energy in numerous configurations but is dominated by those with the lowest energies. To rationalize the *A*_2_ results presented below, we first decomposed the self-interaction energy into contributions from individual residues, averaging over the 1000 lowest energy configurations of WT SUMO. As shown in Figure 4C, two clusters of residues, one basic and one acidic, make major contributions to the self-interaction energy at low pH. The basic residues include H35, K37, K39, H43, K46, and R54 and are on the front face of SUMO (Figure 4A, left); the acidic residues include E67, E83, E84, E64, D86, and E89 and are on the back face (Figure 4A, right).

When the histidines are fully deprotonated, the positive electrostatic potential on the front face of SUMO is reduced (Figures 4A and S8A). Correspondingly, the self-interaction energy overall is weakened, as are the residue-specific contributions (Figure S8B). Upon deprotonation, H35 and H43 suffer the greatest losses in contribution to self-interaction, along with E67, E83, and D86 (Figure S8C), but Lys and Arg residues in the basic cluster maintain significant electrostatic attraction (Figures 4C and S8B).

**Figure 5:**
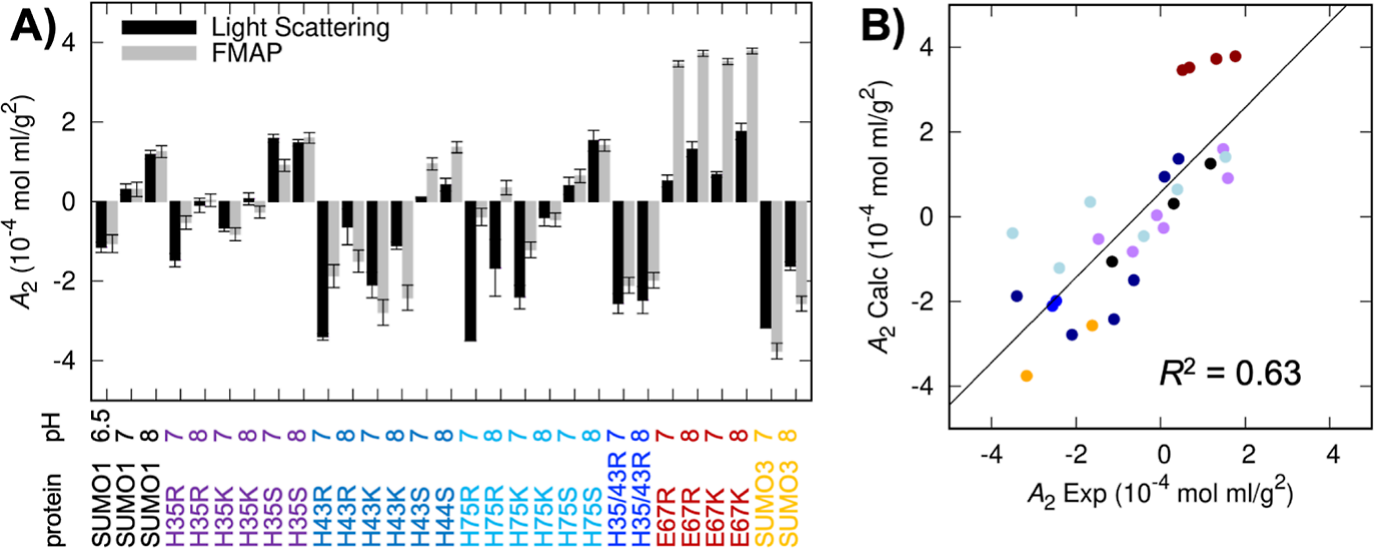
Comparison of calculated and experimental SUMO *A*_2_values. (A) Bar graphs showing calculated *A*_2_ values (FMAP) of the closed conformation of SUMO and those measured by static light scattering. (B) Linear correlation analysis of experimental and calculated *A*_2_ (using the N-tail closed conformation of SUMO; *R*^2^ = 0.63). This *R*^2^ is higher than that for the N-tail open conformation (*R*^2^ = 0.51; Figure S11). Symbol colors are the same as protein names shown in panel (A).

Applying FMAP on the closed conformation of SUMO, we obtained *A*_2_ values at pH 6.5, 7, and 8. To correct for the unknown, but putatively constant, contribution of the flexible N- and C-terminal residues that were not included in the calculations (residues 1–13 and 98–101, respectively), we added a constant to all calculated values to match the predicted and experimental values at pH 7. The predicted values at pH 6.5 and 8 both agree with the experimental counterparts within error (Figure 5A).

We next applied FMAP to the nine single histidine mutants reported in Figure 3 and double mutant H35R/H43R in Figure S7 as well as SUMO3, at both pH 7 and pH 8. As shown in Figure 5A, the agreement between the predicted and experimental *A*_2_ values is generally good.

In all cases, FMAP captures the more positive *A*_2_ values at pH 8 relative to pH 7, as to be expected from weakened electrostatic attraction at the higher pH. It also captures the more positive *A*_2_ values of the Ser mutants than their Arg and Lys counterparts, again attributable to a loss of electrostatic attraction for a neutral residue relative to a basic residue. At a finer level, it accurately predicts that the H35 mutants generally have less negative *A*_2_ values in comparison to the corresponding H43 mutants. The one site that is less well predicted is H75, where the computations indicate that the H75R mutant should have nearly neutral *A*_2_ values at both pH values, whereas the experimental values are highly negative. The behaviors of the H75K mutant are predicted better, however. The underprediction for H75R and H75K is to be expected, given the small computed contribution of H75 to the self-interaction energy of WT SUMO (Figure 4C).

FMAP also correctly predicts the more negative *A*_2_ values of SUMO3 than those of SUMO (Figure 5A). As detailed in Figure S9, the difference arises from the more negatively charged and larger acidic patch in the former isoform.

We also sought to further validate the FMAP calculations through the design of additional mutations. Consistent with the large contribution of E67 to SUMO self-interaction (Figure 4C), FMAP predicted *A*_2_ values for E67R and E67K mutants of approximately 3.5 × 10^–4^ mol mL g^–2^ at pH 7 and a somewhat higher value at pH 8 (Figure 5A). The measured *A*_2_ values of these proteins (Figures 5A and S10A) are indeed among the most positive for all SUMO mutants, though not as positive as the predicted values.

Assessing all the FMAP predictions against the experimental *A*_2_ values by linear regression, a coefficient of determination (*R*^2^) of 0.63 is obtained, along with a slope of unity (Figure 5B). Results calculated using the open conformation of SUMO are worse, with *R*^2^ reducing to 0.51 and the slope (at 1.5) deviating from unity (Figure S11), suggesting that the N-tail is closed during the self-association of SUMO. Overall, these results show that FMAP is a computational tool that can predict, with reasonable accuracy, changes in *A*_2_ that arise from small molecular perturbations such as pH or site-directed mutagenesis.

### Computations Can Reveal Dominant Orientations of Self-Association

**Figure 6:**
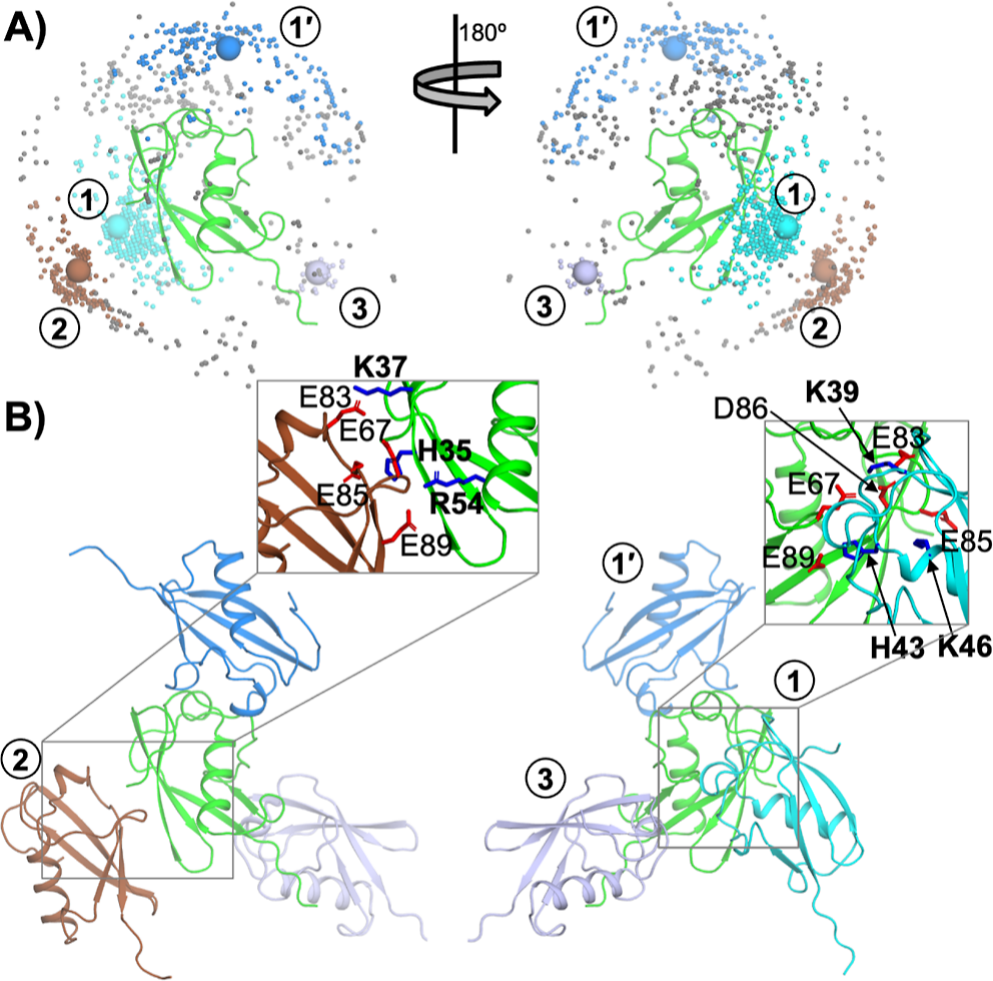
Lowest-energy binary complexes of SUMO. (A) 1000 lowest-energy configurations of SUMO pairs, aligned to one molecule and with the partner molecule represented as a dot located at its center of geometry. The configurations are grouped into four clusters. The two largest clusters, 1 and 1′, are related to each other by a switch of the labels for the two molecules in an asymmetric homodimer. A representative in each cluster is shown as a sphere and displayed as structure in panel (B). (B) Representative structures of the four clusters. Cluster 1, with a close-up view into the interface shown in the inset, has K39, H43, and K46 interacting with the acidic cluster. Cluster 2, with a zoomed-in view into the interface shown in the inset, has H35, K37, and R54 interacting with the acidic cluster.

In addition to energetics, FMAP can also provide an insight into the structural basis of the interactions underlying *A*_2_. As indicated by the residue-specific decomposition, the interaction energy across the protein surface is highly anisotropic, similar to other folded domains.^23,24,42,44^Figure 6A shows the distribution of the 1000 lowest energy poses for a SUMO pair at low pH, with one molecule fixed at the center and the second molecule represented by a dot at its center of geometry. The poses fall into three major clusters (colored in cyan, marine blue, and brown and labeled 1, 1′, and 2, respectively). Clusters 1 and 1′ have similar poses, differing only in which molecule is in the central position (with the other molecule represented as a dot). Figure 6B displays a representative structure from each cluster (sphere in Figure 6A), with expanded views of two of the interfaces shown in the inset. Clusters 1 and 2 involve the acidic patch (centered around E67) of one molecule contacting two different parts of the basic patch separated by the N-tail. Cluster 1 has H43, K39, and K46 in the interface, while cluster 2 has H35, K37, and R54 in the interface. There is also a minor cluster 3 involving the extended C-terminus of SUMO (colored lavender).

When H35 and H43 are deprotonated (at high pH) and hence lose importance, the low-energy poses change orientations. In particular, K16 in the N-tail gains importance energetically (Figure S8) by shifting into the interface with the acidic patch. A similar change in orientation also occurs in SUMO3 upon increasing pH (Figure S9C,D).

Thus, the structural models show that the interactions between the basic and acidic surfaces of SUMO are responsible for self-association of the protein and dictate its *A*_2_ values. The interactions are stronger when the histidines are protonated, accounting for the pH dependence of *A*_2_.

### Changes in SUMO *A*_2_ Mostly Parallel Changes in PolySUMO–PolySIM Phase Separation

**Figure 7:**
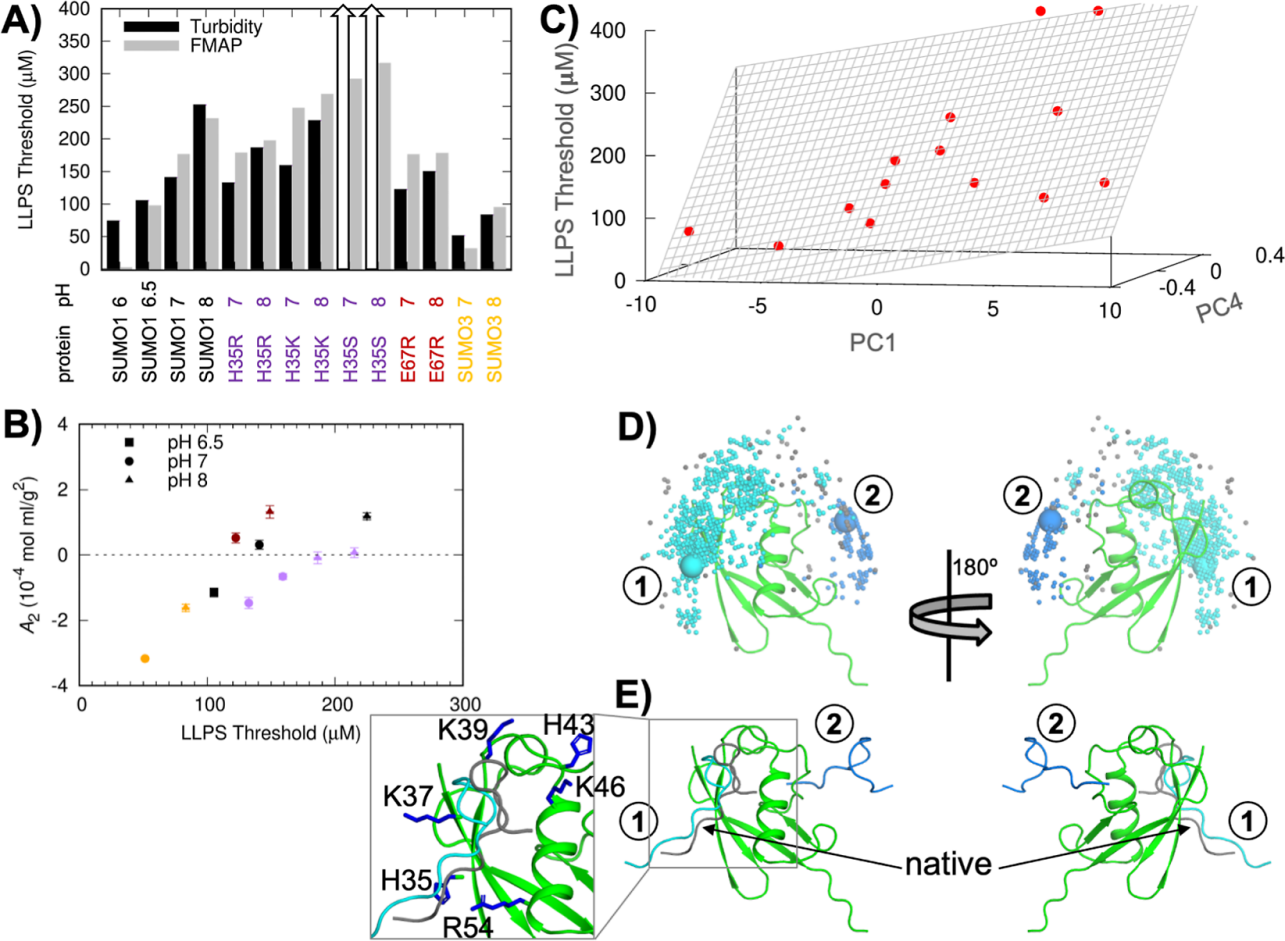
Phase-separation threshold concentrations and lowest-energy weak complexes of SUMO and SIM. (A) Bar graph showing threshold concentrations measured by turbidity, compared with those predicted by multilinear regression of FMAP results. The arrows for the H35S mutant indicate that the protein did not phase-separate up to 400 μM, the highest concentration tested. (B) Correlations between measured *A*_2_ values and phase-separation thresholds for SUMO proteins. Symbol colors are the same as protein names shown in panel (A). (C) Dependence of the measured threshold concentration on two principle components, PC1 and PC4. The plane displays the result of a multilinear regression, given by equation *y* = *a* × PC1 + *b* × PC4 + *c*, with *a* = 6.1, *b* = 413.4, and *c* = 182.5. (D) 1000 lowest-energy configurations of the SUMO–SIM complex determined by FMAP, with SUMO in ribbon representation and SIM as a dot located at its center of geometry. The configurations are grouped into two clusters. Cluster 1 is dominant. A representative in each cluster is shown as a sphere and displayed as structure in panel (E). (E) Representative structures of the two clusters. Cluster 1, with a close-up view of the interface shown in the inset, is similar to the native complex. The native complex is colored with SIM in gray.

To determine whether altering the *A*_2_ of individual SUMO domains through mutagenesis could change the drive for polySUMO–polySIM phase separation, we generated a series of polySUMO molecules in which each SUMO contained an H35R, H35K, or H35S mutation. Each of these variants was mixed with polySIM in equimolar module concentrations and assessed for phase separation by turbidity measurements at pH 7 and 8 (Figure S12). Consistent with the lower *A*_2_ values for SUMO H35R, the polySUMO variant of this mutant had concentration thresholds for phase separation lower than those of the WT protein at both pH values (Figure 7A). PolySUMO H35K, whose SUMO monomer had *A*_2_ values between those of H35R and WT, had a lower threshold than that of WT at pH 8 but higher at pH 7. At both pH values, the polySUMO H35R phase separated more readily than polySUMO H35K, correlating with the relative *A*_2_ values of their monomers. This behavior is consistent with studies implicating arginine as a more potent driver of phase separation than lysine.^12,14,45−47^ In contrast to the R/K mutants, polySUMO H35S did not phase separate with polySIM at either pH 7 or 8 up to the maximum concentration tested (300 μM module). This behavior was again consistent with the strongly positive *A*_2_ values of SUMO H35S at pH 7 and 8, which were similar to that of WT SUMO at pH 8 (Figure 5A). These differences in phase separation behavior are unlikely to be due to differences in SUMO–SIM binding affinity, as the mutants and WT had *K*_D_ values for SIM that were identical within error at pH 7 and 8, as measured by ITC (with the caveat that some values could not be measured due to a small heat of binding, Figure S13). Finally, we examined phase separation of polySUMO3–polySIM. SUMO3 has more negative values of *A*_2_ than WT and H35R/K SUMOs at both pH 7 and 8 (Figure 5A). Consistent with these differences, the phase-separation threshold concentration of polySUMO3–polySIM was the lowest of all proteins examined at both pH values. As illustrated in Figure 7B, the combined data on all proteins at both pHs show a reasonable correlation (*R*^2^ = 0.56) between the *A*_2_ of SUMO monomers and the threshold concentration of polySUMO–polySIM phase separation.

The correlation between *A*_2_ and the phase-separation threshold is not perfect, however. The E67R mutant had *A*_2_ values slightly higher than WT at pH 7 and 8 (Figure 5A), and it bound SIM with an affinity that was statistically indistinguishable from that of WT (Figure S10B). However, the phase-separation thresholds of polySUMO E67R at both pH values were lower than expected based on the behaviors of the other molecules, especially at pH 8 (Figure 7A). Similarly, the H35K mutant had an *A*_2_ value at pH 7 lower than WT, but its polySUMO variant phase-separated with a higher threshold. These results show that SUMO self-association does not completely account for polySUMO–polySIM phase separation.

### Weak SUMO–SUMO and SUMO–SIM Interactions Both Drive Phase Separation

Interactions between all constituent macromolecules can contribute to phase separation.^48−50^ The homotypic second virial coefficient, *A*_2_, captures weak SUMO–SUMO interactions that likely occur inside polySUMO–polySIM droplets. *K*_D_ measures high-affinity stereospecific binding of SIM to the native SUMO site, a configuration that dominates at μM concentrations. At the 1.5–3 mM concentration inside polySUMO–polySIM droplets,^27^ alternate modes of SUMO binding to SIM can also occur. These are of low affinity but have many different configurations and thus make a significant entropic contribution to the binding free energy.^51^ The cross-interaction second virial coefficient, *A*_23_, captures all interactions between SUMO and SIM, involving both the high-affinity native site and the low-affinity additional sites.^44^ Unlike *A*_2_, *A*_23_ is difficult to directly measure experimentally because assay readouts are typically dominated by the high-affinity interactions. However, we have implemented FMAP to calculate *A*_23_.^44^ We note that because FMAP is not designed to optimize high-affinity interactions, it does not single out and reproduce precisely the SUMO–SIM native complex structure but does effectively sample the range of structural contacts between the molecules formed at high concentrations.

The residue-specific decomposition for the cross-interaction between SIM and SUMO in its open conformation at low pH is shown in Figure 4D. A cluster of SUMO basic residues, including H35, K37, K39, H43, K46, and R54, which are also prominent in the self-interaction energy of SUMO, contributes the most to the cross-interaction energy with the highly acidic SIM peptide. However, the acidic cluster that is prominent in the self-interaction energy of SUMO does not contribute significantly to the cross-interaction with SIM. The residue-specific decomposition data for the SUMO-SIM cross-interaction may explain why *A*_2_ and the phase-separation threshold are not correlated in some perturbations (i.e., the E67 mutant present in the acidic cluster in SUMO).

Potentially both SUMO self-interaction and SUMO–SIM cross-interaction may contribute to the drive for polySUMO–polySIM phase separation. SUMO could adopt both the closed (i.e., N-tail bound) and open conformations in these interactions. Therefore, a combination of *A*_2_ and *A*_23_ values calculated for the closed and open conformations may explain the phase-separation threshold data. The *A*_2_ and *A*_23_ data calculated for the different pH values and various mutants are correlated because they involve the same basic cluster containing the histidine residues. Thus, instead of directly using the *A*_2_ and *A*_23_ data for multilinear regression against the phase-separation threshold data, we first carried out a principal component (PC) analysis on the *A*_2_ and *A*_23_ data themselves (Figure S14A). Two of the resulting orthogonal PCs, PC1 and PC4 (Figure S14B), are highly correlated with the phase-separation threshold in a multilinear regression (*R*^2^ = 0.73; Figure 7C). Predicted threshold concentrations based on the regression are in good agreement with the experimental data (Figure 7A). In comparison, regression using only the calculated *A*_2_ values produced only a modest correlation (*R*^2^ = 0.27). The improved agreement, particularly for the polySUMO E67R mutant, suggests that *A*_23_ also contributes to phase separation. It remains unclear what the residual deviations between the experiment and model derive from. One likely source of deviation is the fact that modeling was performed only on individual SUMO and SIM proteins, whereas these modules are tethered by flexible linkers in polySUMO and polySIM. While the linkers are not charged, they may nevertheless restrict orientations of the domains in ways that influence charge effects on phase separation in a manner that is specific to the different mutants and/or conditions.

Most poses with low cross-interaction energy between SIM and SUMO in the open conformation and at low pH fall into a single cluster (colored cyan and labeled 1 in Figure 7D). This cluster includes the native pose (at lower resolution; Figure 7E) but spreads over a broad region, where the acidic residues of SIM contact the basic cluster on the front side of SUMO. There is also a minor cluster (colored marine blue and labeled 2 in Figure 7D) around SUMO R70 on the back side.

## Conclusions

**Figure 8:**
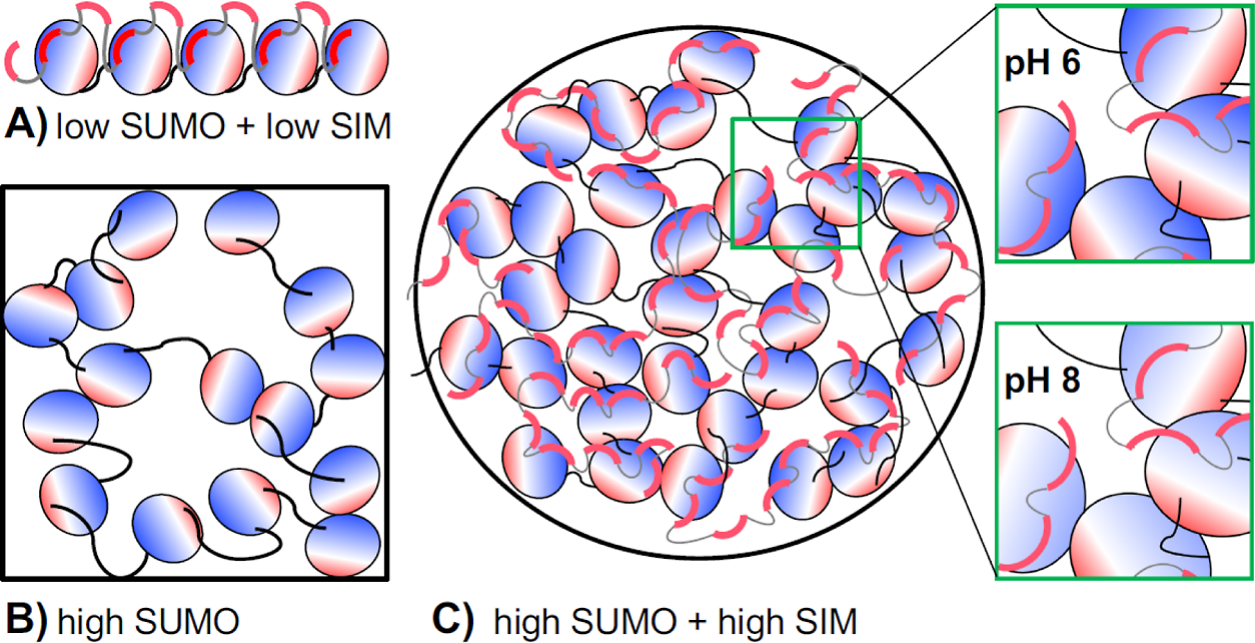
Illustration of intermolecular interactions. (A) At low concentrations of polySUMO and polySIM, SIM only occupies the high-affinity site on SUMO. (B) When polySUMO is concentrated, SUMO modules interact with each other by pairing oppositely charged surface patches. (C) When polySUMO and polySIM are mixed at high concentrations (as found inside droplets), SIM occupies a multitude of low-affinity sites in addition to the specific site on SUMO. SUMO modules also interact with each other, as found in a concentrated polySUMO solution. The insets show that these weak interactions are stronger at low pH due to greater charge on the positive surface patch of SUMO, increasing the drive to phase-separate. Due to the covalent linkages between the modules, polySUMO and polySIM form highly interconnected molecular networks.

We have shown here that the phase separation of the polySUMO–polySIM system is strongly pH dependent. This property does not arise from changes in SUMO–SIM binding affinity as affinity is pH independent. Rather, experiments and modeling suggest that it arises from pH dependence of weak SUMO–SUMO self-association and weak SUMO–SIM cross-association (through sites not sampled by the high-affinity interaction), assessed by *A*_2_ and *A*_23_, respectively. Both types of weak interactions involve surface-exposed histidines that change protonation states with pH, thus rendering phase separation sensitive to pH. In driving phase separation, high-affinity, stereospecific SUMO–SIM interactions generate large oligomers, dependent on the valence and affinity of the interactions and distance constraints on geometry introduced by the linkers.^52,53^ This oligomerization decreases the intrinsic solubility of the molecules, potentiating phase separation entropically.^31,54−57^ Weak, nonstereospecific SUMO–SUMO and SUMO–SIM interactions also decrease the solubility of SUMO monomers and oligomers and thus increase the drive for phase separation. As shown in Figure 8, high-affinity stereospecific SUMO–SIM binding is important at concentrations around *K*_D_ (∼μM) (Figure 8A). At the high concentrations (1–3 mM) inside phase-separated droplets, weak interactions between SUMO domains (Figure 8B) and between SUMO and SIM enable diverse binding species to be populated and rapidly interconvert (Figure 8C).

Importantly, the strength of the weak interactions can be tuned by pH via alteration of the protonation state of surface histidines (Figure 8C inset). The electrostatic potential around a basic cluster on SUMO that includes histidines is more intense when the imidazole moieties are protonated at pH 6 than when they are deprotonated at pH 8. Correspondingly, the electrostatic interactions with an acidic patch on a folded domain or with the acidic residues of SIM are stronger at pH 6 than at pH 8. The measured p*K*_a_ values of the three histidine side chains are between 6.3 and 6.5 (Figure S5), values consistent with a surface-exposed imidazole group on a folded domain and lacking an ion pair,^58^ corresponding to partial protonation at each site of 16–24% at pH 7 and 2–3% at pH 8. When occurring in the polySUMO array, this difference can evidently produce substantial changes in the phase separation behavior. Electrostatic interactions along surfaces of protein domains driving phase separation have been previously reported for a naturally occurring protein, SHP2.^59^ This system, along with our findings with polySUMO, suggests that electrostatic interactions from folded domains contributing to phase separation may be widely observed.

Computational methods, which apply experimental and theoretical knowledge to predict the molecular determinants of phase separation, have been used to engineer phase separation behaviors in a number of systems. Numerous computational tools have been developed that successfully predict the phase separation behavior of IDRs based on amino acid sequence.^12,60−64^ However, none of these tools are suitable to identify the weak adhesive elements on the surface of folded protein domains that define their solubility and drive phase separation. FMAP was the first method to predict the phase diagram of folded protein domains based on atomistic modeling of protein–protein interactions.^23^ Subsequently, FMAP was also implemented to calculate *A*_2_ and *A*_23_, quantifying the weak self-association or cross-association of folded domains, by enumerating the interaction energy between two molecules across all separations and orientations.^42,44^ We show here that this information is also useful in predicting phase-separation. Justification for using *A*_2_ and *A*_23_ as predictors of the phase separation threshold concentration is provided by recent work showing that phase-separation equilibrium properties such as the critical temperature are correlated with the virial coefficients.^24^ A unique feature of FMAP is that it can provide a structural picture of the most energetically favorable interactions between two weakly associating molecules. For SUMO, FMAP predicted that H35 and H43 contact the acidic surface of neighboring SUMO molecules, suggesting a likely mechanism for pH-dependent SUMO self-association. It also predicted that favorable nonspecific SIM binding occurs in the neighborhood of the stereospecific site. We note that while FMAP was reasonably accurate in quantitative predictions of *A*_2_ of SUMO and its variants, it was highly accurate in predicting the sign of *A*_2_. Future computational tools designed to predict the phase separation behavior of complex molecules consisting of folded domains and IDRs would benefit from a unified view that considers weak adhesions from both domain surfaces and disordered elements.

We envision several means by which the surface properties of folded domains in multidomain proteins could be modulated to control phase separation in both biological and engineering settings and on different time scales. Charge could be altered in vivo over evolutionary time scales through changes to surface residues, independent of sites that mediate high-affinity binding to ligands. Relatedly, disease mutations could also act by modifying charged surface residues, altering higher order assembly/phase separation of molecules and consequent functions. Surface charges could be changed in real time in vivo through post-translational modifications (PTMs) such as phosphorylation or acetylation. The effects of PTMs on the phase separation of IDRs have been widely observed. Our work suggests that similar behaviors might be observed for folded domains. In engineering applications, surface charges of folded domains could be used to tune the phase-separation threshold of multidomain systems and potentially the material properties of the droplets that they produce. It may also be possible to use surface variants to explore the potential functional differences between molecular network formation and phase separation. For example, the H35S mutant should retain the capacity to create large molecular networks held together through SUMO–SIM interactions without undergoing a density transition characteristic of phase separation. In contrast, the H35R and WT proteins should create networks similarly but also phase separate. These species could thus be used to examine whether network formation and phase separation have different effects on the macromolecular function. Overall, our observations suggest new means of controlling phase separation and its functional consequences in vitro and in vivo.

## Materials and Methods

### Monomeric SUMO1 and SUMO3 Expression and Purification

All SUMO domain proteins were expressed with an N-terminal His_8_-tag in BL21(DE3) T1R cells in TB media, collected by centrifugation, and lysed using cell disruption (Emulsiflex-C5, Avestin) in a buffer containing 20 mM Tris-HCl (pH 8.0), 300 mM NaCl, 1 mM PMSF, 1 μg/mL antipain, 1 μg/mL pepstatin, and 1 μg/mL leupeptin. The lysate was clarified by centrifugation, and the supernatant was applied to Ni-NTA agarose resin (Qiagen). The resin was first washed with a buffer of 20 mM Tris-HCl, 300 mM NaCl, and 20 mM imidazole (pH 8.0) and with a second wash with a buffer of 20 mM Tris-HCl, 150 mM NaCl, and 30 mM imidazole (pH 8.0). Protein was eluted in 20 mM Tris-HCl, 150 mM NaCl, and 250 mM imidazole (pH 8.0). The His_8_-tag was removed using TEV protease at 4 °C overnight. The cleaved protein was applied to a Source15Q (Cytiva Life Sciences) anion exchange column and eluted using a gradient from 0 to 500 mM NaCl in 20 mM Tris-HCl (pH 8.0), 1 mM EDTA, and 1 mM DTT. Fractions containing SUMO were pooled and further purified using a Superdex200 prepgrade column (Cytiva Life Sciences) in 20 mM HEPES, 150 mM KCl, 1 mM MgCl_2_, 1 mM DTT, and 1 mM EGTA (pH 7.0).

### PolySUMO1 and PolySUMO3 Expression and Purification

MBP-polySUMO-His_6_ and MBP-polySUMO3-His_6_ and their mutants were expressed and purified through Ni-NTA agarose (Qiagen) identically to the SUMO monomers (see above). Protein eluted from this resin was applied to amylose resin (New England Biolabs) and washed with a buffer consisting of 20 mM Tris-HCl, 150 mM NaCl, 1 mM EDTA, and 1 mM DTT (pH 8.0). Bound protein was eluted with this same buffer but also containing 52 mM maltose (pH 8.0). The His_6_ and MBP tags were removed using TEV protease at 4 °C overnight. The cleaved protein was further purified using Source15Q and Superdex200 prepgrade columns identically to the SUMO monomers (see above).

### Static Light Scattering

Samples for light scattering were buffer-exchanged using a Superdex75 Increase 10/300 gel filtration column (Cytiva Life Sciences) into 20 mM HEPES, 150 mM KCl, 1 mM MgCl_2_, 1 mM DTT, and 1 mM EGTA (pH 7.0 or 8.0). Fractions with protein were concentrated to ∼200 μL, and the solution was ultracentrifuged at 100,000 g for 1 h at 4 °C. The top 100 μL of the sample was used for light scattering studies. These procedures were necessary to obtain high-quality data. All light scattering measurements were performed using a Wyatt DyanPro NanoStar light scattering instrument at 25 °C. Immediately prior to measurement, the sample was diluted using buffer to the appropriate concentration, and any remaining particulates were removed by centrifugation at 21,000 g for 5–10 min. For each measurement, 2.2 μL was added to a JC-562 quartz cuvette and equilibrated inside the instrument for at least 5 min prior to data acquisition. At each concentration, three measurements were made using 10 scans over 60 s each. The second virial coefficient (*A*_2_) was determined from the slope of a linear fit of scattering intensity versus concentration using Dynamics software. *A*_2_ was independently measured three times, and the averages and the standard deviations are reported.

### Protein Structure Preparation for FMAP Calculations

The structure of SUMO1 was from PDB entry 1Y8R chain C, with residues 14–97 representing the closed conformation and residues 21–97 representing the open conformation. The extreme terminal residues were not included due to their flexibility. The structure for the core region of SUMO3, i.e., residues 16–86 (which align to SUMO1 residues 21–91; see Figure S9B), was from PDB entry 2IO1 chain B. SUMO3 residues 9–15 and 87–92 were modeled by homology modeling^65^ using 1Y8R as a template. In analogy to SUMO1, SUMO3 residues 9–92 represented the closed conformation, and residues 16–92 represented the open conformation. The structure of SIM was from PDB entry 2ASQ chain B, residues 1–14. Given the potential flexibility of SIM, we carried out FMAP calculations using SIM structures from the different NMR models in 2ASQ. The results from the different models were similar, and we report those calculated on model 1.

PDB2PQR^66^ was used to add hydrogens and assign AMBER charges and radii, producing the pqr file. Mutations were also generated in this step by deleting the coordinates of the residue under mutation and introducing the name of the mutated residue. Asp and Glu were deprotonated, whereas Lys and Arg were protonated. For His residues, all possible combinations of protonation states (eight for wild-type SUMO1 with three His residues and four for wild-type SUMO3 with two His residues) were enumerated, and the results at a given pH were based on a Boltzmann average over all the possible protonation states (see below).

### FMAP Calculations and Related Analyses

The second virial coefficient, *A*_2_, was calculated by the FMAP method, which was previously implemented into a web server: https://pipe.rcc.fsu.edu/fmapb2/.^42^ The input was the pqr file, with ionic strength set to 0.175 M and temperature set to 298 K. Similarly, the cross second virial coefficient, *A*_23_, modeling the weak interaction between two different protein molecules (e.g., SUMO and SIM), was calculated using a web server: https://pipe.rcc.fsu.edu/fmapb23/.^44^

The interaction energies of the 1000 lowest-energy configurations identified by the FMAP method were further analyzed using an atom-based method, which entailed enumerating interactions over all pairs of atoms.^67^ The total interaction energy in each configuration was decomposed into the contributions from individual residues. The contribution of each residue was then averaged over the 1000 lowest-energy configurations. The decomposition was done for both self-interaction (after the *A*_2_ calculation) and cross-interaction (after the *A*_23_ calculation).

The 1000 configurations were clustered according to the ligand-rmsd. Ligand rmsd is the root-mean-square deviation between two poses of the smaller partner after superposition of the larger protein. We used a ligand rmsd cutoff of 9 Å to define clusters. All configurations within the ligand rmsd cutoff of any existing member of a cluster were collected for that cluster. Clusters were retained only when they had at least 10 members.

### Boltzmann Average over His Protonation States

We considered all possible protonation states of the His residues. Each His residue was assigned a p*K*_a_ value of 6.3 (Figure S5), and its probability for being protonated or deprotonated was calculated according to the Henderson–Hasselbach equation. For a protein with three His residues, there are eight possible combinations of protonation states. *A*_2_ or *A*_23_ was calculated for each combination of protonation states and then Boltzmann-averaged over all the combinations of protonation states, with the Boltzmann weight for each combination given by the product of the probabilities for the protonation states of the individual His residues.

### Principal Component Analysis of *A*_2_ and *A*_23_ Results

We used the *A*_2_ and *A*_23_ results calculated on both the open and closed conformations of SUMO or SUMO3 to predict the phase-separation threshold concentration based on multiple linear regression (MLR). The four sets of results, *A*_2_ from open conformation, *A*_2_ from closed conformation, *A*_23_ from open conformation, and *A*_23_ from closed conformation, are correlated. The correlations posed problems for both the quality of the regression and the interpretation of the results. To circumvent these problems, we first subjected the input data to principal component analysis (PCA), which yields PCs that by definition are orthogonal (hence correlation-free).

The input to the PCA was four sets of *A*_2_ and *A*_23_ data for 30 combinations of protein variant and pH (see also Figure S14 legend). We then applied the PCA module from the scikit-learn library (https://scikit-learn.org), which implements a singular value decomposition of the input data. The four PCs extracted were used for the trial MLR. We finally settled on using only PC1 and PC4, as the other two PCs contributed little to the coefficient of determination.

### Linear Regression Analysis

Linear regression was carried out using the R package (https://www.r-project.org/). Simple linear regression, e.g., between calculated and measured *A*_2_ (Figures 5B and S11), called function lm(*y* ∼ *x*); MLR called function lm(*y* ∼ *x*1 + *x*2). For polySUMO H35S, the measured threshold concentrations were assumed to be 400 μM in the MLR.

### ITC Measurements

SUMO and SIM were buffer-exchanged using a Superdex75Increase 10/300 or Superdex Peptide 10/300 gel filtration column (Cytiva Life Sciences) into 20 mM HEPES, 150 mM KCl, 1 mM MgCl_2_, 1 mM DTT, 1 mM EGTA (pH 7.0 or 8.0) or 20 mM MES, 150 mM KCl, 1 mM MgCl_2_, 1 mM DTT, and 1 mM EGTA (pH 6.5). ITC experiments were performed using a Microcal ITC200 isothermal calorimeter using 50–100 μM SIM peptide in the cell and 750–1.2 mM SUMO in the syringe. Baseline corrections and integrated heats were performed using NITPIC.^68^ Affinity was determined using a 1:1 binding model in SEDPHAT.^69^ Reported error is the standard deviation from the Monte Carlo simulation based on the experimental noise.

### NMR Experiments

WT SUMO1 was exchanged into a buffer consisting of 20 mM phosphate, 20 mM citrate, 150 mM KCl, 1 mM MgCl_2_, 1 mM DTT, and 1 mM EGTA at a pH of 5.5, 6.0, 6.33, 6.67, 7.0, 7.33, 7.67, or 8.0 using a Superdex75Increase 10/100 column, respectively. Samples were concentrated to 0.5–0.8 mM WT SUMO1. The ^1^H/^13^C HSQC spectra were acquired on a 600 MHz Varian Inova II spectrometer using a spectral window of 43 ppm centered at 125 ppm using 1024 × 128 complex points, 16 transients, and a recycling delay of 1 s. All experiments were performed at 25 °C in 8% D_2_O buffer. NMR data were processed using NMRpipe^70^ and visualized using Sparky.^71^ The data were fit to a modified form of the Hill equation using nonlinear regression
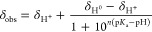
where δ_obs_ is the observed histidine ^1^Hε or ^13^Cε chemical shift at the measured pH and δ_H^+^_ and δ_H^0^_ are the chemical shifts for the protonated and neutral forms of the histidine imidazole ring, respectively. Reported errors are the 95% confidence intervals as given by the nonlinear fit.

### Phase Diagram Mapping

PolySUMO and polySIM proteins were buffer-exchanged using a Superdex200Increase 10/300 gel filtration column (Cytiva Life Sciences) into 20 mM HEPES, 150 mM KCl, 1 mM MgCl_2_, 1 mM DTT, 1 mM EGTA (pH 7.0 or pH 8.0) or 20 mM MOPS, 150 mM KCl, 1 mM MgCl_2_, 1 mM DTT, and 1 mM EGTA (pH 6.5). 2 μL of polySUMO and 2 μL of polySIM proteins were mixed together on the pedestal of a NanoDrop OneC spectrophotometer (Thermo Scientific). After 2–3 min, the absorbance at 340 nm was measured. For each module concentration, the measurement was performed in triplicate. The phase-separation threshold concentration was determined from the *x*-axis intercept of a line fitted to the first three points with *A*_340_ > 0.1.

## Supporting Information Available

Light scattering, ITC, and NMR data and their respective analysis; FMAP calculations and analysis; and PCA of FMAP results (PDF)

## Abbreviations

IDR: intrinsically disordered region
SLIM: short linear interaction motif
SUMO: small ubiquitin-like modifier protein 1
SIM: SUMO interacting motif
FMAP: fast Fourier transform-based modeling of atomistic protein–protein interactions
ITC: isothermal titration calorimetry
