# Supplementary material for: Surface Charge Can Modulate Phase Separation of Multidomain Proteins
Source: J Am Chem Soc. 2024 Jan 23;146(5):3383–95. doi: 10.1021/jacs.3c12789 (PMC10859935; doi:10.1021/jacs.3c12789)
Supplement: Supplementary file 1 — ja3c12789_si_001.pdf [file ja3c12789_si_001.pdf]

Supporting Information

Surface charge can modulate phase separation of multi-domain proteins

Jonggul Kim[1,2], Sanbo Qin[3], Huan-Xiang Zhou[3,4], Michael K. Rosen*[1,2]

[1]Department of Biophysics, University of Texas Southwestern Medical Center and [2]Howard Hughes Medical Institute, Dallas, Texas 75390

[3]Department of Chemistry and [4]Department of Physics, University of Illinois at Chicago, Chicago, Illinois 60607

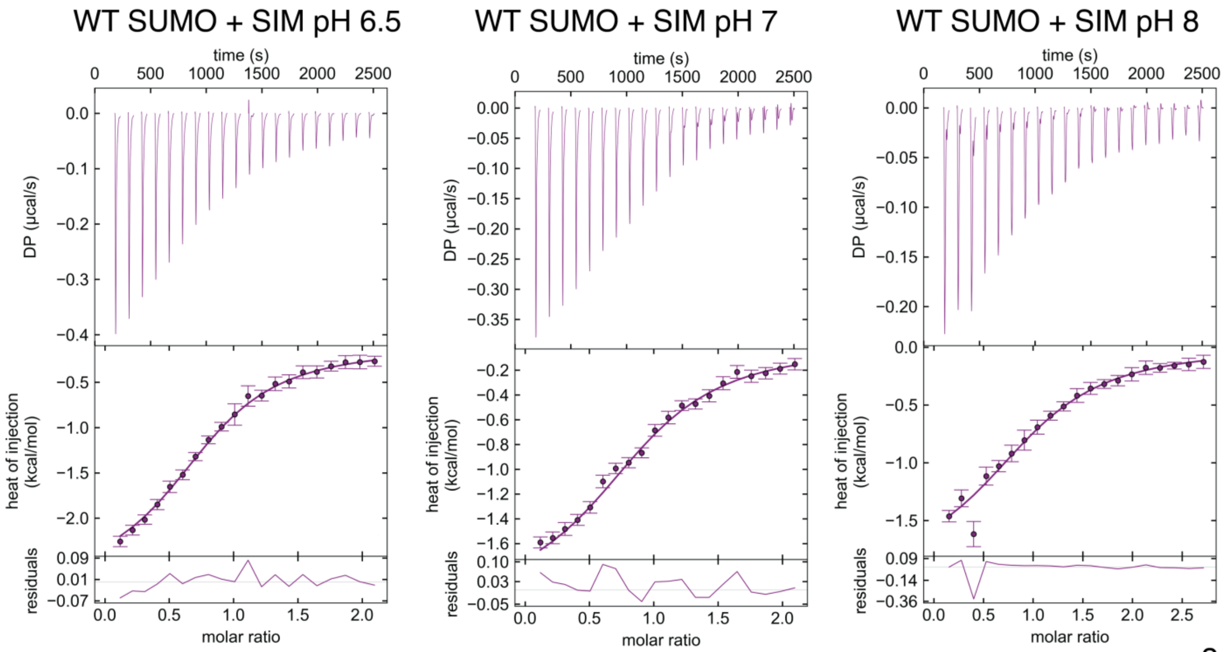

Figure S1: Binding of SIM to WT SUMO as measured by ITC at pH 6.5, 7.0, and 8.0.

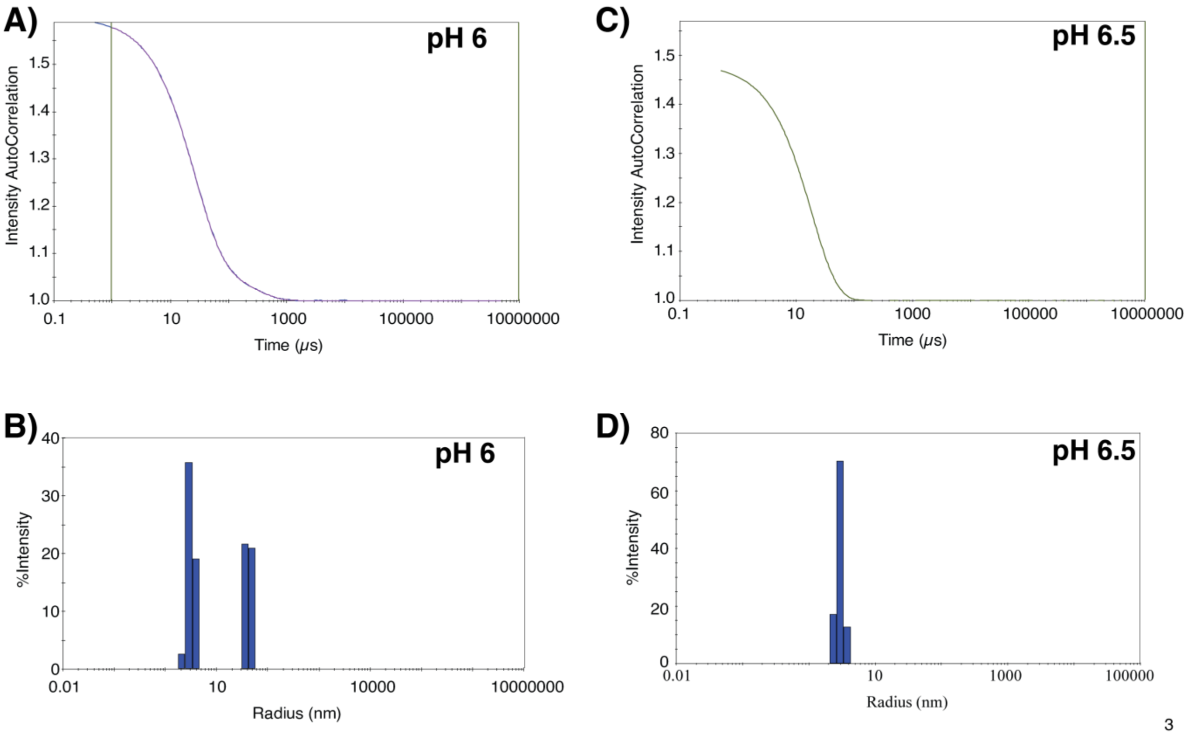

Figure S2: WT SUMO makes large soluble aggregates in solution at pH 6. A) Dynamic light scattering autocorrelation plot and B) the regularization graph of the scattering autocorrelation of WT SUMO at pH 6. Note that WT SUMO has a large scattering intensity around a radius of ~ 100 nm at pH 6. C) and D) Corresponding results at pH 6.5, demonstrating that this aggregate is not present at the higher pH.

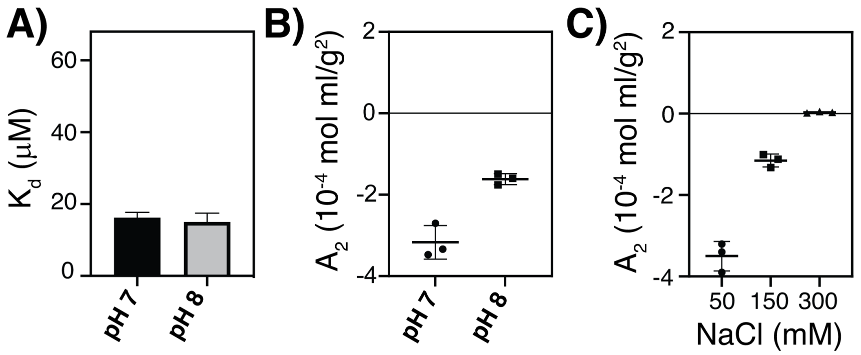

Figure S3: pH only affects A_2, not the affinity of SIM for the SUMO3 isoform; and salt dependence of SUMO1 A_2 values. A) K_d measured between WT SUMO3 and SIM at pH 7 and 8 by using ITC. The affinity is identical within error, indicating that the ability to form networks is unchanged with pH. B) The A_2 of WT SUMO3 at pH 7 and 8 determined by static light scattering, indicating a change in weak self-association with pH. C) The A_2 of WT SUMO1 at pH 6.5 is dependent on salt concentration, suggesting that the interaction is electrostatic in nature.

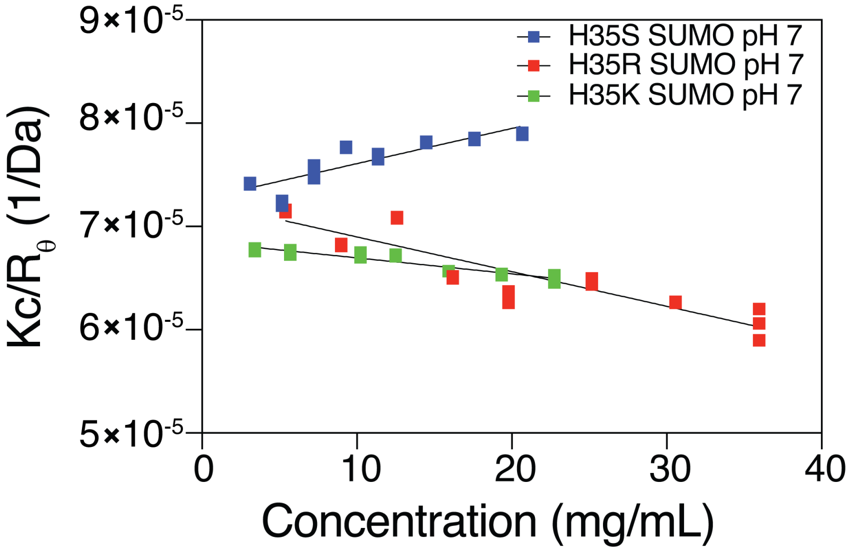

Figure S4: Static light scattering data for SUMO mutants at pH 7 as a function of concentration.

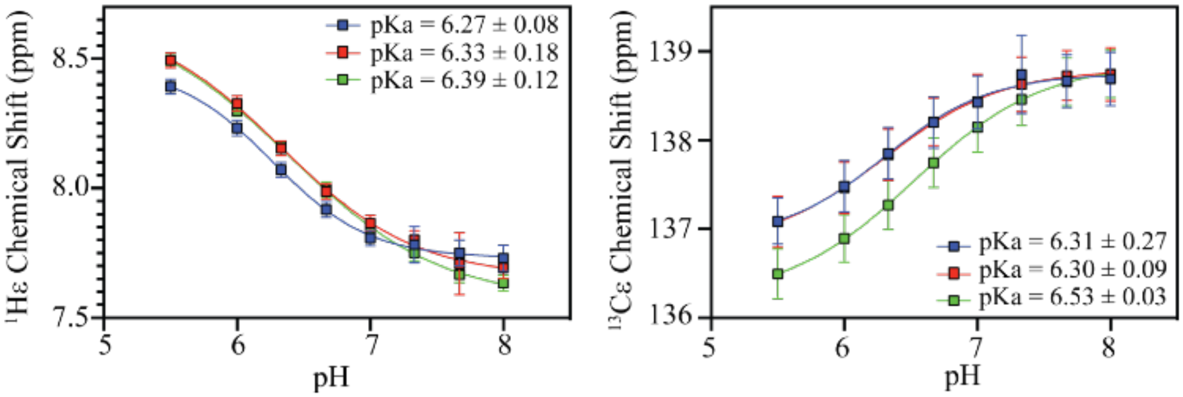

Figure S5: pH titration of WT SUMO assessed by NMR spectroscopy. A) pH dependence of the ^{1}Hε chemical shifts and B) the pH dependence of the ^{13}Cε chemical shifts. The fitted pKa values are shown in the inset of the plot. Although sequence specific chemical shift assignments are not available for these moieties, all three histidine ^{1}Hε and ^{13}Cε signals in the NMR spectra had measured pKa values between 6.27 and 6.53, and are identical for each histidine within experimental error.

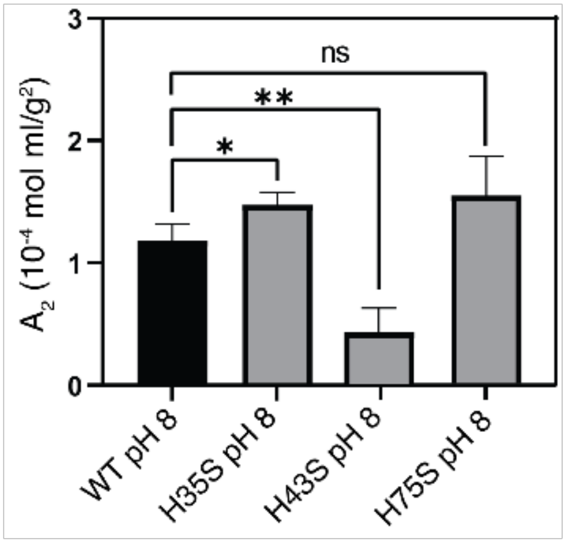

Figure S6: T-test of the histidine to serine mutations at pH 8 compared to WT SUMO. The t-test shows that the H35S and H43S mutations caused a statistically significant change of A_2 ($p < 0.05$ for H35S and $p < 0.01$ H43S), while the H75S mutation did not cause a statistically significant difference in A_2.

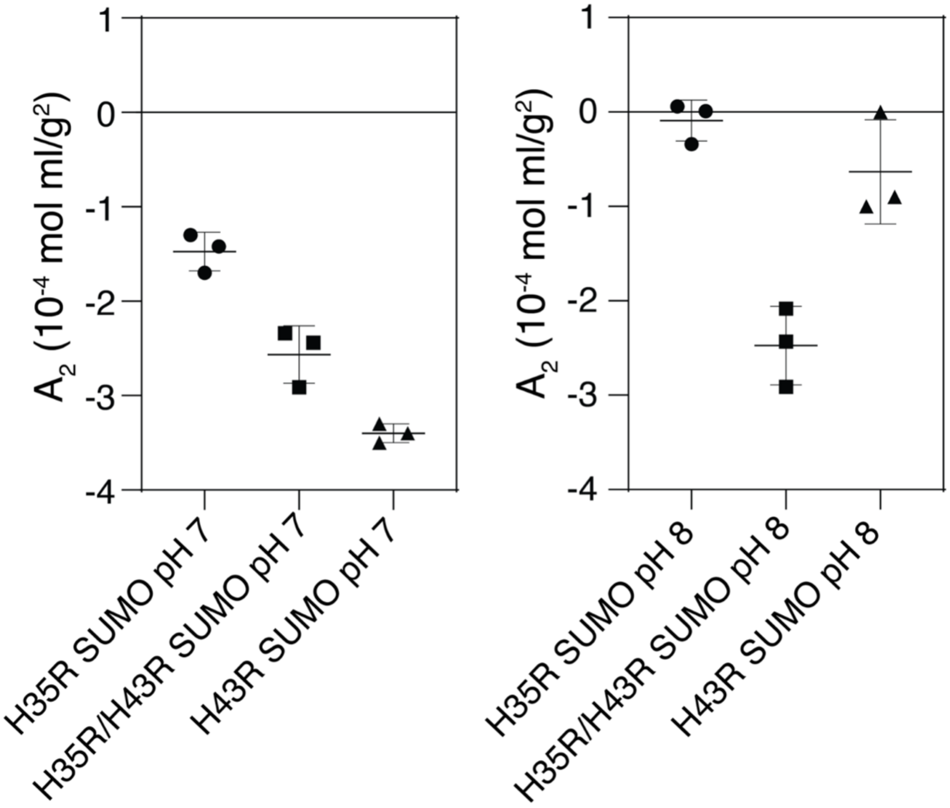

Figure S7: Second virial coefficients of the SUMO H35R/H43R double mutant at pH 7 (left) and pH 8 (right). Values for the SUMO H35R and H43R single mutants are included for comparison.

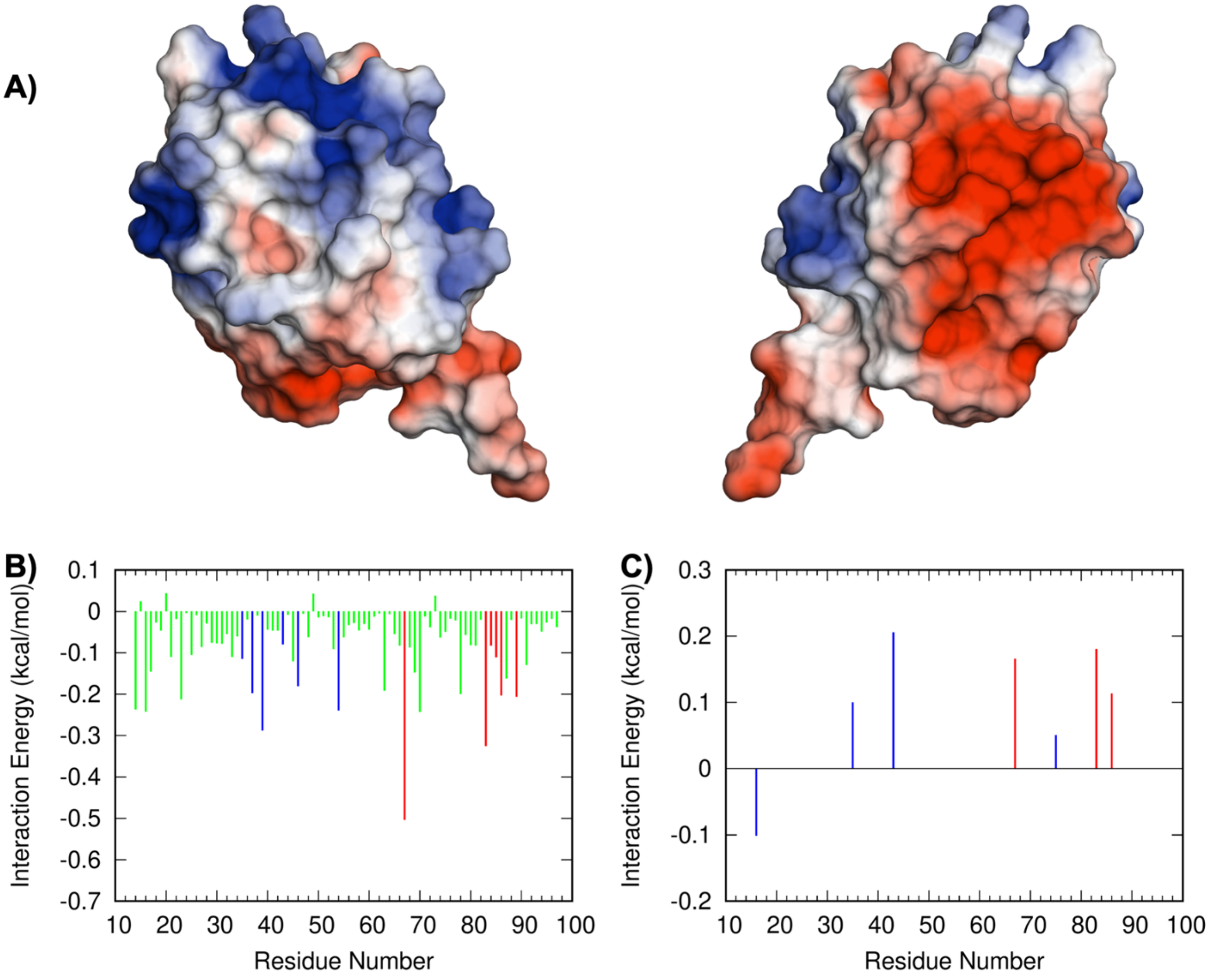

Figure S8: Results similar to those presented in Fig. 4, but calculated at high pH, i.e., with all His residues deprotonated. A) Surface electrostatic potential of SUMO1. B) Decomposition of the binary self-interaction energy of SUMO. Values for prominent basic residues (H35, K37, K39, H43, K46, and R54) and acidic residues (E67, E83, E84, E85, D86, and E89) are shown as blue and red bars, respectively. C) The difference between high pH and low pH results for inter-residue interactions. Residues showing the highest pH dependence are indicated by blue (K16, H35, H43) or red (E67, E83, and D86) bars; H75 is also shown by a blue bar.

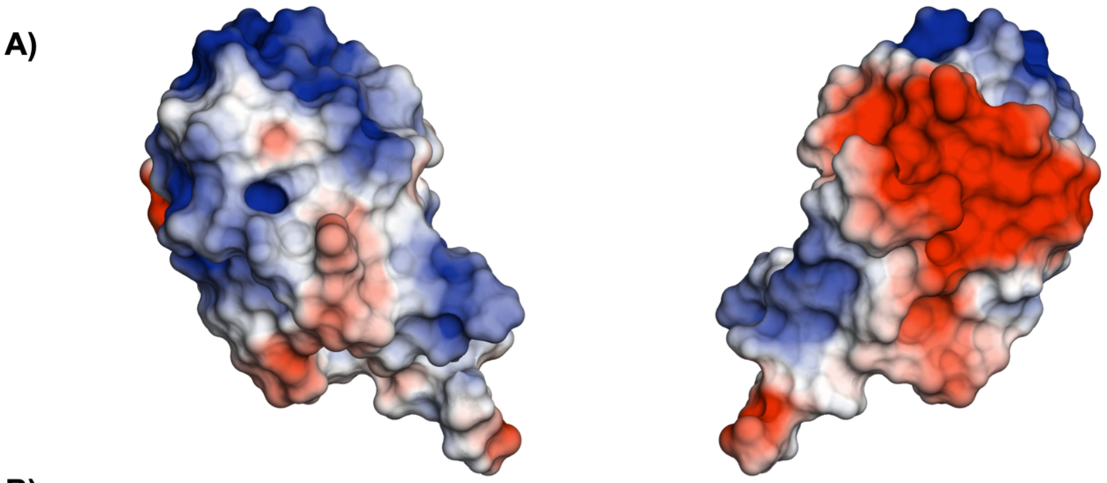

B)

```
SOMO1 14     GDKKEGE YIKLKVIGQD SSEIHFKVKM TTHLKKLKES YCQRQGVPMN 60
SUMO3  9     GVKTEND HINLKVAGQD GSVVQFKIKR HTPLSKLMKA YCERQGLSMR 55

SUMO1 61 SLRFLFEGQR IADNHTPKEL GMEEEDVIEV YQEQTGG 97
SUMO3 56 QIRFRFDGQP INETDTPAQL EMEDEDTIDV FQQQTGG 92
```

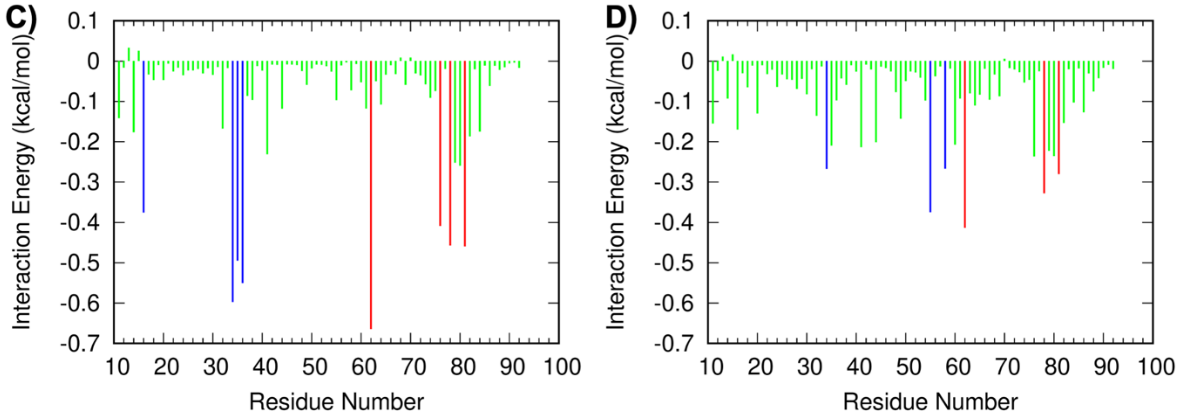

Figure S9: Results similar to those presented in Fig. 4, but for SUMO3. A) Surface electrostatic potential of SUMO3 calculated at low pH, i.e., with all the His residues protonated. The negative potential on the back face is more intense and spreads more widely than the SUMO1 counterpart (**Figure 4A**, bottom right), due to substitutions to acidic residues (D70 and E76 in SUMO3). B) Sequence alignment of SUMO1 and SUMO3. Conserved basic and acidic residues are shown in blue and red, respectively; His residues are in green. C) Decomposition of the binary self-interaction energy of SUMO3 at low pH. The highest contributions are shown as blue bars (H16, K34, R35, and H36) or red bars (D62, E76, E78, and D81). Basic residues including H16, H36, K34, and R35 of SUMO3 make greater contributions to self-interaction than the prominent basic residues in SUMO1 (**Figure 4C**). D) Same as panel C but calculated at high pH. At high pH, H16 and H36 lose importance; instead, other basic residues, R55 and R58, gain prominence.

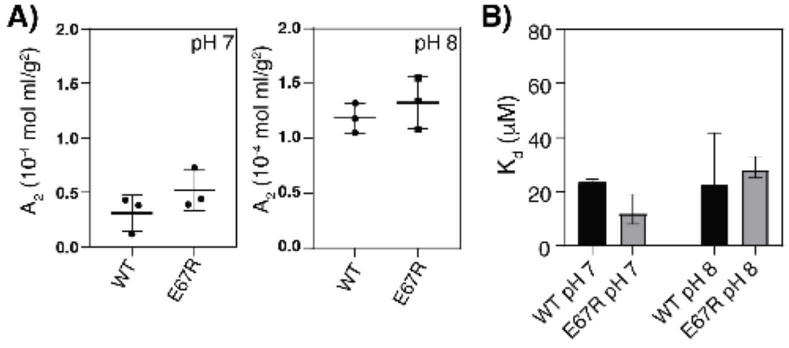

Figure S10: Change in binding affinity and weak self-association of the SUMO E67R mutant. A) The A_2 of E67R is higher than that of WT SUMO and B) the binding affinities of SUMO E67R for SIM at pH 7 and 8 are similar to those of WT SUMO for SIM.

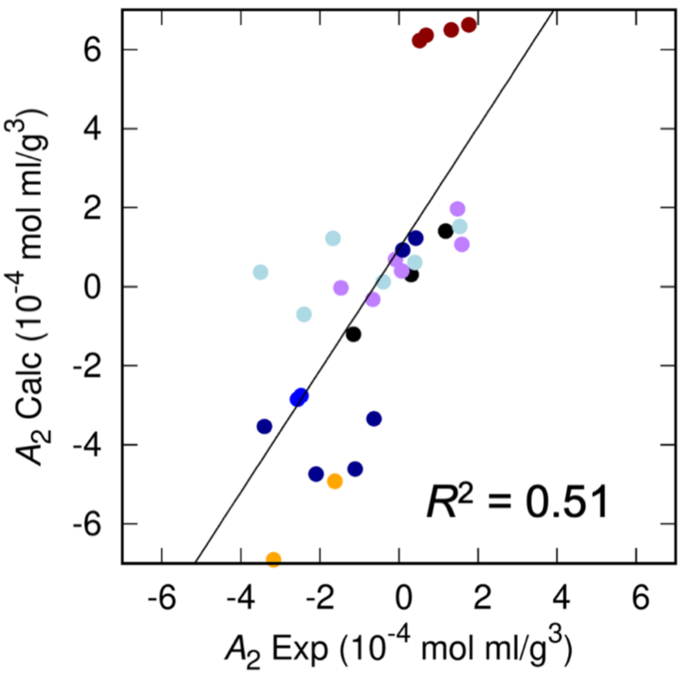

Figure S11: Linear correlation analysis of calculated and experimental results for A_2 values of SUMOs at different pHs and with a variety of mutations. The calculated results here assumed the N-tail open conformation of SUMO. Symbols colors are: SUMO1, black; SUMO3, yellow; H35 mutants, purple; H43 mutants, dark blue; H75 mutants, cyan; H35/H43 double mutants, royal blue; and E67 mutants, red.

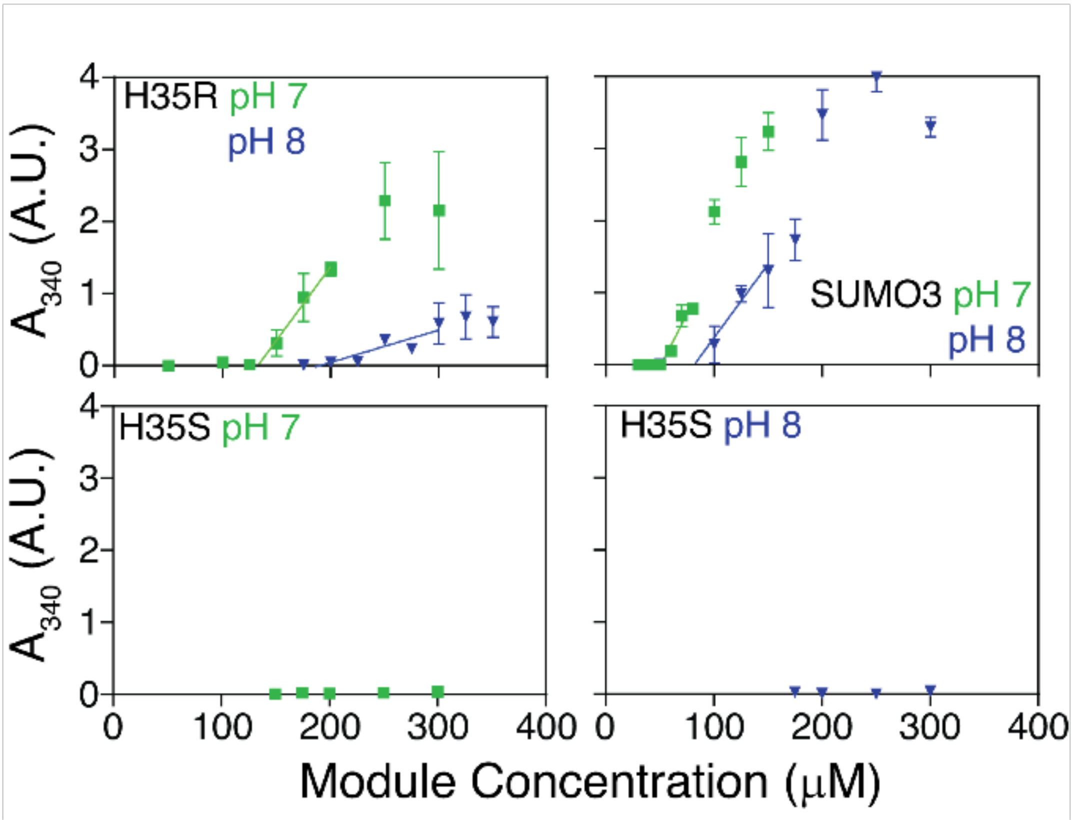

Figure S12: Turbidity measurements at 340 nm for mutant polySUMO and polySUMO3 titration with an equal module concentration of polySIM at pH 7 (green) and 8 (blue). The extrapolation to determine the phase separation threshold was performed by calculating a linear fit from the first three concentrations with an $A_{340} > 0.1$.

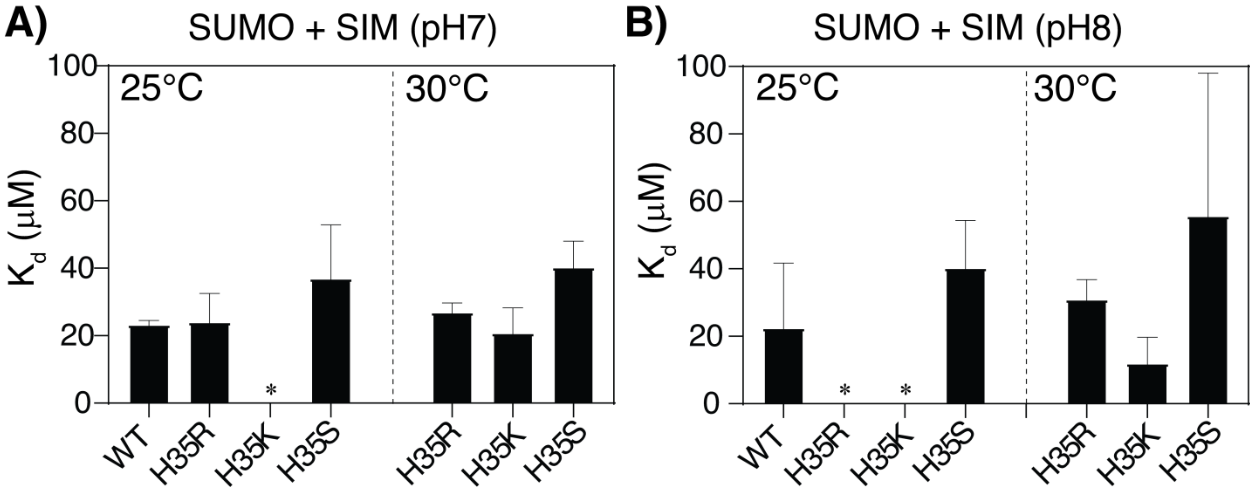

Figure S13: The affinities of SUMO mutants for SIM measured by ITC at A) pH7 and B) pH 8. Asterisks indicate that no heat was detected at the indicated temperature. ITC binding experiments did not show substantial deviation in measured affinity between 25°C and 30°C.

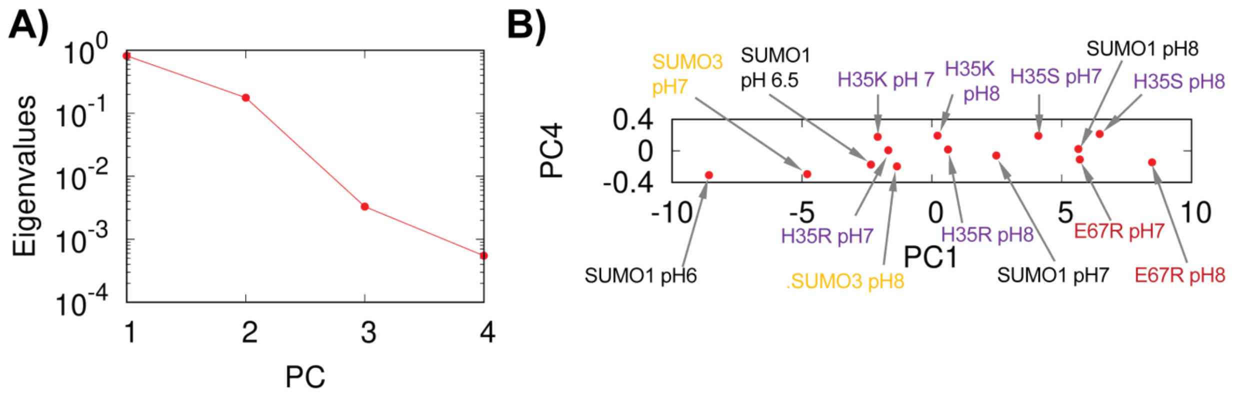

Figure S14: Principal component analysis of FMAP results. A) Eigenvalues of four sets of FMAP results. Each set of results comprised A_2 or A_{23} values calculated using the open or closed conformations of SUMO, for 30 combinations of protein variant and pH (29 listed in Fig. 5 plus SUMO at pH 6). The four orthogonal eigenvectors have normalized amplitudes in the range of 0.3 to 0.7 along the four virial coefficients. PC1 has positive values for all its four components, while the higher PCs each have two positive and two negative components. In particular, PC4 has positive components along A_2 closed and A_{23} open. B) Projection of the FMAP results for 14 combinations of protein variant and pH along two of the principal components, PC1 and PC4. These 14 combinations were those for which phase-separation threshold concentrations were measured. The two PCs were selected because they showed the highest correlations with the measured threshold concentrations.
